# Safety and efficacy of prophylactic and therapeutic vaccine based on live-attenuated *Listeria monocytogenes* in hepatobiliary cancers

**DOI:** 10.1038/s41388-022-02222-z

**Published:** 2022-02-16

**Authors:** Inga Hochnadel, Lisa Hoenicke, Nataliia Petriv, Lavinia Neubert, Elena Reinhard, Tatjana Hirsch, Juan Carlos Lopez Alfonso, Huizhen Suo, Thomas Longerich, Robert Geffers, Ralf Lichtinghagen, Carlos Alberto Guzmán, Heiner Wedemeyer, Henrike Lenzen, Michael Peter Manns, Dunja Bruder, Tetyana Yevsa

**Affiliations:** 1 Department of Gastroenterology, Hepatology and Endocrinology, Hannover Medical School (MHH), Hannover, Germany; 2 Institute of Pathology, MHH, Hannover, Germany; 3 Department of Vaccinology and Applied Microbiology, Helmholtz Centre for Infection Research (HZI), Braunschweig, Germany; 4 Immune Regulation Group, HZI, Braunschweig, Germany and Infection Immunology Group, Institute of Medical Microbiology and Hospital Hygiene, Otto-von-Guericke University Magdeburg, Magdeburg, Germany; 5 Department of Systems Immunology, Technical University Braunschweig and HZI, Braunschweig, Germany; 6 Institute of Pathology, Heidelberg University Hospital, Heidelberg, Germany; 7 Genome Analytics, HZI, Braunschweig, Germany; 8 Department of Clinical Chemistry, MHH, Hannover, Germany

**Keywords:** Liver cancer, Immunotherapy

## Abstract

Primary liver cancer (PLC) comprising hepatocellular carcinoma (HCC) and cholangiocarcinoma (CCA) represents the third deadliest cancer worldwide with still insufficient treatment options. We have previously found that CD4 T helper 1 (Th1) response is indispensable for the protection against PLC. In the present research, we aimed to test the potent inducers of Th1 responses, live-attenuated *Listeria monocytogenes ∆actA/∆inlB* strain as preventive/therapeutic vaccine candidate in liver fibrosis, HCC, and CCA. Studies were performed using autochthonous models of HCC and CCA, highly reflecting human disease. *L. monocytogenes ∆actA/∆inlB* demonstrated strong safety/efficacy in premalignant and malignant liver diseases. The protective mechanism relied on the induction of strong tumor-specific immune responses that keep the development of hepatobiliary cancers under control. Combination therapy, comprising Listeria vaccination and a checkpoint inhibitor blockade significantly extended the survival of HCC-bearing mice even at the advanced stages of the disease. This is the first report on the safety and efficacy of Listeria-based vaccine in liver fibrosis, as well as the first proof of principle study on Listeria-based vaccines in CCA. Our study paves the way for the use of live-attenuated Listeria as safe and efficient vaccine and a potent inducer of protective immune responses in liver fibrosis and hepatobiliary malignancies.

## Abstract (web summary)

Protective immune mechanism induced by live-attenuated double-deleted *L. monocytogenes ∆actA/∆inlB* vaccine strain delivering tumor antigens keeps hepatobiliary malignancies under control. Live-attenuated, double-deleted *L. monocytogenes ∆actA/∆inlB* strain expressing model tumor antigen was used in both, prophylactic and therapeutic vaccination settings. Vaccination was safe and led to: (i) induction of protective tumor-specific Th1 immune responses in premalignant and malignant stages and strong increase of tumor-specific IFN-ɣ^+^ CD4 and CD8 T cells; (ii) decrease of T regulatory cells; (iii) downregulation of several tumor-promoting ICI molecules (PD-1, CD160, LAG3, 4-1BBL) on CD4/CD8 T lymphocytes; (iv) decrease of tumor-specific IgG in serum, and (v) decrease of B lymphocytes, DC and MΦ locally in livers. iCCA, intrahepatic cholangiocarcinoma.

## Graphical abstract

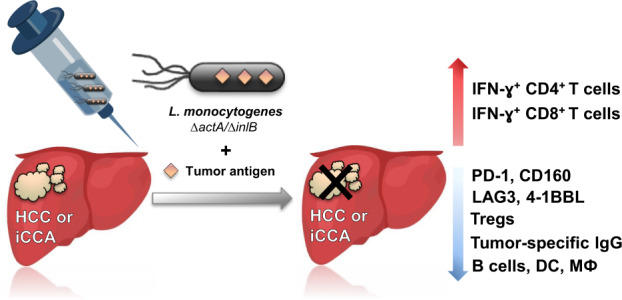

## Introduction

Primary liver cancer (PLC) is the third deadliest cancer worldwide encountering 830,000 deaths per year [1, 2]. Hepatocellular carcinoma (HCC) and intrahepatic cholangiocarcinoma (CCA) represent the most abundant PLC types [3]. Treatment options for HCC and CCA include curative resection, liver transplantation for HCC, locoregional and systemic therapies [4, 5]. Unfortunately, most cases present with advanced unresectable disease and systemic therapies are still associated with a poor outcome in both PLC subtypes.

For patients with unresectable HCC, sorafenib has been the only systemic therapeutic with proven clinical efficacy since 2007 [6]. Recently, the immunotherapy combination of atezolizumab (inhibitor of programmed death-ligand 1 (PD-L1)) and bevacizumab (inhibitor of vascular endothelial growth factor) demonstrated an extended median progression-free and overall survival compared to sorafenib and has been approved by the Food and Drug Administration (FDA) [7]. Trials with immune checkpoint inhibitors (ICIs) revealed very promising data, currently pembrolizumab and nivolumab (inhibitors of PD-1) are approved by the FDA as a second-line treatment in advanced HCC [8]. Further, several phase II and III trials are investigating different combination therapies with various ICIs and tyrosine kinase inhibitors in advanced HCC [9, 10].

While chemotherapy still remains the gold standard for unresectable CCA, several promising trials are currently under evaluation for triplet regimens, different immunotherapeutic agents, and targeted therapies. However, due to the molecular complexity of CCA, many initial approaches have revealed unsatisfactory results [10]. Although new therapies for HCC and CCA in the palliative setting are emerging, treatment options are still very limited and there is an urgent need for improved innovative treatment and prevention strategies.

In previous studies, we have shown that induction of a T helper 1 (Th1)-polarized CD4 T cell response is indispensable to prevent PLC development [11–14]. In patients with PLC, T cell responses are strongly impaired, due to a highly suppressive tumor milieu and a reactivation of tumor-specific Th1 CD4 and CD8 T cell responses is indispensable to mediate control of liver cancer progression [10].

Therefore, in the present research, we focused on live-attenuated *Listeria monocytogenes* strains as delivery platforms of cancerous antigens, and potent inducers of strong anti-tumor Th1 CD4 and CD8 T cells responses in different solid cancers, as reviewed [10, 15]. To test the suitability of live-attenuated *L. monocytogenes* vaccination as a potential prophylactic or curative treatment for PLC, we used the double deficient strain *L. monocytogenes ∆actA/∆inlB* (designated as LmAI), lacking the actin assembly-inducing protein (ActA) and internalin B (InlB). LmAI is incapable of cell-to-cell spread and displays decreased capacity to invade hepatocytes [16]. To be able to follow-up and characterize the anti-tumor immune responses induced via vaccination, we took advantage of a LmAI derivative delivering the model antigen Ovalbumin (Ova), designated as LmAIO. Further, we investigated the preventive/therapeutic vaccination capacity of LmAIO and a protective mechanism beyond in autochthonous HCC and CCA, both co-expressing the tumor model antigen Ova, and in liver fibrosis.

## Materials and methods

Section can be found in *Supplementary Information*.

## Results

### Autochthonous mouse models of HCC-Ova and CCA-Ova

**Fig. 1:**
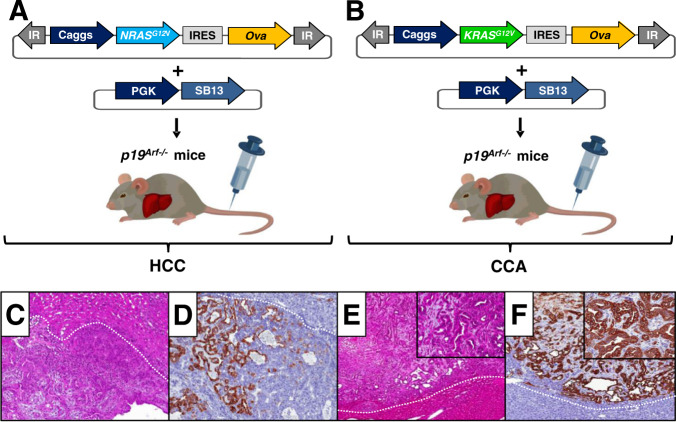
Vectors and experimental design. **A**, **B** *NRAS*^*G12V*^*-Ova* (HCC-Ova) (**A**) or *KRAS*^*G12V*^*-Ova* (CCA-Ova) (**B**) transposon constructs were co-delivered with SB13 transposase into p19^Arf−/−^ mice using HDI. **C–F** H&E and CK7 immunohistochemical staining of mouse livers injected with *NRAS*^*G12V*^*-Ova* (**C**, **D**) and *KRAS*^*G12V*^*-Ova* (**E**, **F**). Dotted line indicates border of the tumor. Magnification x 100 (**C**, **D**), x 50 (**E**, **F**), inserts of x 200 (**E**, **F**). H&E, haematoxylin and eosin; IR, inverted repeats; pCaggs, CAG promoter and PGK, phosphoglycerate kinase promoter, described in our previous studies [11, 14, 19, 28]. IRES, internal ribosome entry site; SB13, Sleeping Beauty 13 transposase.

To induce HCC-Ova and CCA-Ova development, we stably delivered transposable elements co-encoding *NRAS*^*G12V*^*-Ova* or *KRAS*^*G12V*^*-Ova*, respectively, into hepatocytes of p19^Arf−/−^ mice via hydrodynamic tail vein injection (HDI) (Fig. 1A, B). P19^Arf−/−^ mice lack the tumor suppressor Arf and stable intrahepatic delivery of *RAS*^*G12V*^ oncogenes co-expressed with Ova into p19^Arf−/−^ hepatocytes resulted in multinodular autochthonous tumor formation [11, 17–19]. Mice injected with *NRAS*^*G12V*^-*Ova* developed HCC and occasionally combined HCC-CCA as demonstrated by cytokeratin 7 (CK7)-positive ductal areas (Fig. 1C, D), whereas intrahepatic delivery of *KRAS*^*G12V*^-*Ova* resulted in the formation of predominantly CK7^+^ CCA (Fig. 1E, F).

In both models (HCC-Ova and CCA-Ova) multiple neoplastic foci were observed as early as 7 days after HDI (Supplementary Table S1). Thus, for therapeutic vaccination day 7 has been selected as “early tumor stage” time point. Days 14 and 21 were considered as “intermediate and advanced tumor stages”, respectively (Supplementary Table S1).

### Live-attenuated LmAIO strain is safe for in vivo application

Two live-attenuated *L. monocytogenes* vaccine strains, LmAI, and its counterpart expressing Ova (designated as LmAIO), were used for in vivo application. Listeria was administered intravenously (*i.v*.) at a dose corresponding to the established 0.1 LD_50_ [16]. In line with published data [16], 24 h after vaccination highest Listeria counts were detected in liver and spleen of mice while significantly lower numbers were found in pancreas and lung and only a few bacteria were detected in blood (Supplementary Fig. S1A). At sampling, neither bacteria, nor significant histopathological changes were observed in the analyzed organs after vaccination with LmAIO (Supplementary Fig. S1B-E). Interestingly, focal accumulations of pigmented macrophages were present in liver parenchyma (Supplementary Fig. S1F-H), suggesting phagocytosis of Listeria. Furthermore, the first exposure to LmAI and LmAIO caused only a minor weight loss followed by a fast recovery in p19^Arf−/−^ and wild type (WT) mice (Supplementary Fig. S1I, J and the experimental conditions are described in the next section) correlating with previous reports [15, 16]. No weight loss was detectable upon booster vaccination (Supplementary Fig. S1I, J, day −7).

Taken together, LmAI and LmAIO were safe for in vivo administration.

### Prophylactic vaccination with LmAIO reduces HCC and induces pronounced tumor-specific Th1 immune responses thereby decreasing tumor-specific IgG

**Fig. 2:**
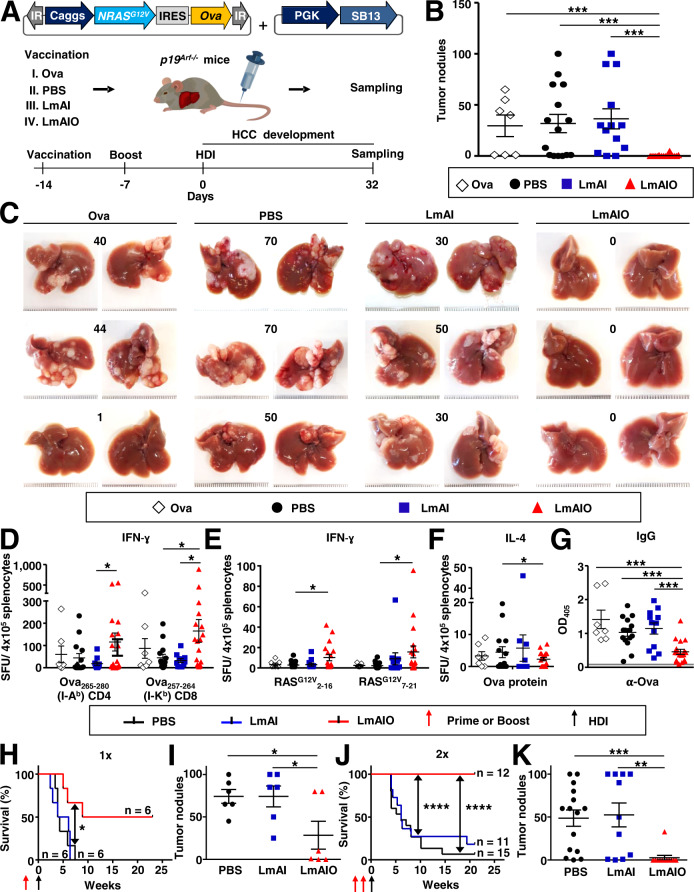
Prophylactic immunization with live-attenuated LmAIO induces tumor antigen-specific Th1 responses, mediates reduction of HCC burden and prolongs survival of mice. **A** Experimental setup to study the prophylactic potential of LmAIO in HCC-Ova settings. **B**, **C** Quantification of macroscopic HCC nodules (**B**) and representative photographs of explanted livers with corresponding tumor burden at sampling (**C**). **D**, **E** IFN-ɣ analysis on splenocytes from p19^Arf−/−^ mice after re-stimulation with CD4- and CD8- specific Ova peptides (**D**) and oncogenic RAS^G12V^ peptides (**E**). **F** IL-4 ELISPOT analysis of splenocytes after re-stimulation with LPS-free Ova protein. **G** ELISA to detect Ova-specific IgG in serum of p19^Arf−/−^ mice receiving two prophylactic vaccinations. A gray line represents blank values. **H**–**K**, Kaplan–Meier survival curves and tumor burden in explanted livers of p19^Arf−/−^ mice vaccinated once (**H**, **I**) or twice (**J**, **K**). Numbers of animals per group in (**B**–**G**) (Ova *n* = *7*, PBS *n* = *15*, LmAI *n* = *13*, LmAIO *n* = *18*), in (**H–K**) numbers of animals are depicted in the plots. Data in (**B**–**G**) are pooled from four individual experiments. Data were analyzed using unpaired *t*-test and Mantel–Cox test for survival. *P < 0.05, **P < 0.01, ***P < 0.001, ****P < 0.0001. Shown are mean ± SEM. SFU, spot-forming units; HDI, hydrodynamic tail vein injection.

We first analyzed the preventive potential of the vaccine strain LmAIO in p19^Arf−/−^ and WT mice in comparison to control groups receiving PBS, Ova protein and LmAI. Vaccinations were applied on days −14 and −7 prior to the induction of HCC-Ova (Fig. 2A). Mice were sacrificed 32 days after HCC-Ova induction.

LmAIO-vaccinated mice were entirely protected from HCC-Ova development, whereas multilocular tumor development was detected in all p19^Arf−/−^ control groups (Fig. 2B, C). In line with previous studies, no tumors were detected at sampling in WT mice (data not shown) [11, 19].

To unravel tumor-protective mechanisms induced in LmAIO-vaccinated mice, enzyme-linked immunospot (ELISPOT) assays with CD4 and CD8 Ova peptides, Ova protein, mutant RAS^G12V^-specific peptides and heat-killed/sonicated Listeria (whole cell antigen) were performed. Despite the fact that all experimental groups received HDI with *NRAS*^*G12V*^-*Ova*, only LmAIO-vaccinated group broke the tolerance [16] and demonstrated significant CD4 and CD8 T cell responses against Ova, shown most pronounced in CD8 T cells (Fig. 2D). Re-stimulation of splenocytes with RAS^G12V^ peptides revealed significant RAS^G12V^-specific IFN-ɣ responses in splenocytes of LmAIO-vaccinated animals suggesting epitope spreading [20, 21] induced by the vaccine strain (Fig. 2E). Importantly, IFN-ɣ responses to Ova and RAS^G12V^ antigens detected in p19^Arf−/−^ mice correlated with IFN-ɣ responses in vaccinated tumor-free WT mice (Supplementary Fig. S2A, B). IFN-ɣ responses against Listeria whole cell antigen were only detected in LmAI- and LmAIO-immunized groups (Supplementary Fig. S2C). Interestingly, in p19^Arf−/−^ background LmAIO-vaccinated animals showed significantly lower IFN-ɣ responses towards heat-killed Listeria than LmAI-vaccinated mice. The latter effect was not observed in WT animals (Supplementary Fig. S2C).

In contrast to IFN-ɣ, there was no increase in IL-4 responses (corresponding to Th2 immunity) toward Ova protein, CD4 Ova peptide, and RAS^G12V^ peptides in LmAIO-vaccinated p19^Arf−/−^ and WT animals in comparison to other groups (Fig. 2F, Supplementary Fig. S2D-F).

Analysis of tumor-specific antibodies in vaccinated p19^Arf−/−^ animals at sampling revealed a marked significant decrease in quantity of Ova-specific IgG in LmAIO group (Fig. 2G), while Listeria-specific IgM and IgG antibody responses (α-LmAI and α-LmAIO) were not decreased in LmAIO-vaccinated p19^Arf−/−^ and WT groups (Supplementary Fig. S2G-J).

Taken together, preventive vaccination with LmAIO broke the tolerance, induced a Th1 immune response against the tumor antigen delivered by Listeria and strongly reduced tumor-specific IgG. The induced Ova- and RAS^G12V^-specific Th1 immune responses were comparable in p19^Arf−/−^ and WT mice.

### Prophylactic vaccination with LmAIO fully protects from HCC development

To assess long-term prophylactic capacity of LmAIO vaccination, survival was monitored. Single LmAIO vaccination applied 2 weeks before the induction of HCC-Ova development prolonged survival of 50% of mice compared to PBS or LmAI groups (Fig. 2H). Strikingly, one additional LmAIO boost vaccination rescued all mice for the monitored time period (Fig. 2J). While single LmAIO vaccination was sufficient to significantly reduce hepatic tumor burden (Fig. 2I), a further boost yielded a more pronounced effect with only one out of twelve LmAIO-vaccinated mice developing HCC (Fig. 2K). All survival experiments in this study were abrogated between weeks 20 and 25 after HDI to avoid unspecific tumor development known to occur in aged p19^Arf−/−^ mice [22].

In summary, the preventive LmAIO vaccination significantly increased survival and protected mice from HCC-Ova development in a dose-dependent manner.

### Immunization with Listeria is safe and efficient in mice with liver fibrosis

Precancerous liver diseases like fibrosis or cirrhosis are the major risk factors for HCC progression [5, 23]. Several reports showed that immune responses towards vaccine antigens are not efficient in patients with fibrosis and/or cirrhosis in comparison to patients with healthy livers [24, 25]. We aimed to test whether live-attenuated Listeria delivering tumor model antigen Ova is able to induce strong immune responses against Ova in animals with liver fibrosis and whether this vaccination is safe.

**Fig. 3:**
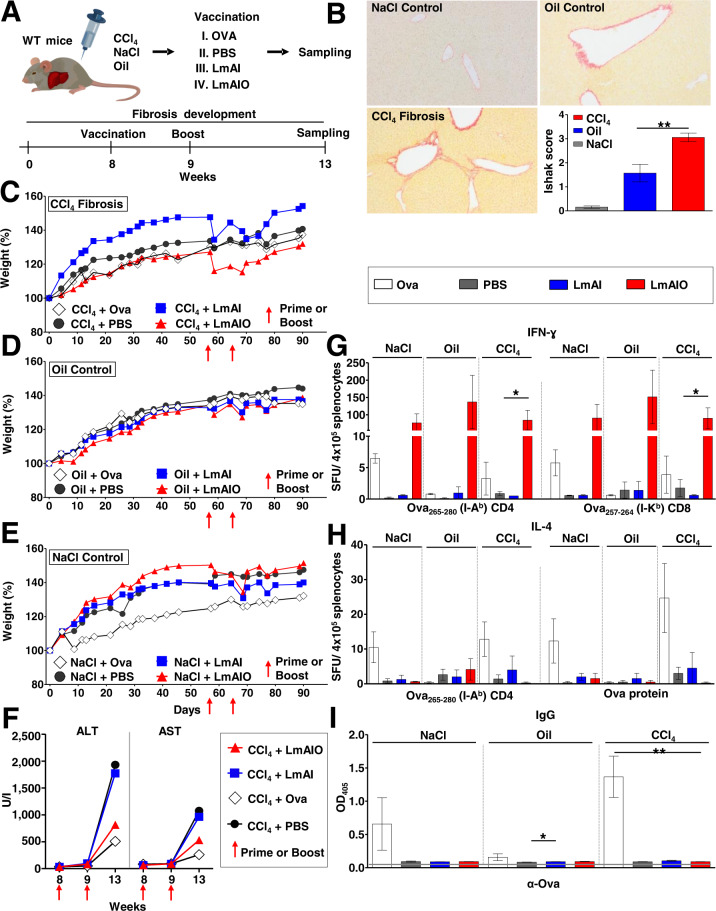
LmAIO vaccine is safe and efficient in mice with fibrotic livers. **A** Experimental setup to study Listeria-based vaccine in fibrosis-bearing mice. **B** Sirius red staining of mouse liver 8 weeks upon NaCl (*n* = *4*), oil (*n* = *7*) or CCl_4_ (*n* = *9*) treatment and corresponding Ishak score demonstrating the severity of fibrosis. (Magnification NaCl control x 40; oil control and CCl_4_ fibrosis x 100). **C**–**E**, Weight development of CCl_4_− (**C**), oil- (**D**) and NaCl-treated mice (**E**). **F** ALT and AST kinetic upon vaccination with Listeria or controls in fibrosis-bearing mice. **G** IFN-ɣ ELISPOT analysis on splenocytes re-stimulated with CD4 and CD8 Ova peptides. **H** IL-4 ELISPOT analysis on splenocytes re-stimulated with either CD4 peptide or Ova full protein. **I** ELISA on Ova-specific IgG in plasma of mice. Numbers of animals in (**C**–**I**) (NaCl + PBS *n* = *3*, NaCl+Ova *n* = *3*, NaCl+LmAI *n* = *2*, NaCl+LmAIO *n* = *2*, oil+PBS *n* = *4*, oil+Ova *n* = *2*, oil+LmAI *n* = *3*, oil+LmAIO *n* = *4*, CCl_4_ + PBS *n* = *4*, CCl_4_ + Ova *n* = *3*, CCl_4_ + LmAI *n* = *2*, CCl_4_ + LmAIO *n* = *4*). Data were analyzed using unpaired *t*-test. *P < 0.05, **P < 0.01. Shown are mean ± SEM. SFU, spot-forming units.

To induce liver fibrosis, animals were treated twice per week with tetrachloromethane (CCl_4_) or respective controls (NaCl or sunflower oil, Fig. 3A, B). Whereas NaCl-treated control mice were free of pathological changes, livers of mice that underwent CCl_4_ treatment (diluted in a carrier (oil)) displayed fibrous changes and occasional bridging with a mean Ishak score of F3 corresponding to fibrosis (Fig. 3B). Livers of oil-treated mice showed a minor fibrous expansion corresponding to a mean Ishak score of F1.6 with minimal fibrosis (Fig. 3B). Upon fibrosis development, two doses of the vaccine, prime and boost, were applied in weeks 8 and 9, and CCl_4_ treatment was continued until sampling (Fig. 3B). Treatment of mice with CCl_4_ did not affect the weight development and only a transient weight loss upon prime or boost vaccination with a fast recovery was observed (Fig. 3C-E). LmAI/LmAIO vaccination did not induce additional liver toxicity shown by the levels of alanine aminotransferase (ALT) and aspartate aminotransferase (AST) in comparison to CCl_4_/PBS control group (Fig. 3F). Strong induction of Ova-specific CD4 and CD8 INF-ɣ responses were detected in all LmAIO-vaccinated groups and they were independent of fibrosis, demonstrating comparable INF-ɣ responses between NaCl, oil, and CCl_4_ groups (Fig. 3G). Strong INF-ɣ responses towards heat-killed Listeria were detected in Listeria-vaccinated groups only and were also fibrosis-independent (Supplementary Fig. S3A). No significant Ova-specific IL-4 responses were detected in Listeria-vaccinated groups (Fig. 3H), which also correlated with Ova-specific IgG responses (Fig. 3I). Listeria-specific IgM and IgG levels were increased in Listeria-vaccinated animals only and were mostly pronounced in fibrosis-bearing animals (Supplementary Fig. S3B-E).

Taken together, vaccination with LmAIO in fibrosis-bearing mice was safe, did not exacerbate liver damage and induced strong Th1 immune responses against the antigen delivered by Listeria. The latter responses were comparable to those obtained in mice with healthy livers.

### Therapeutic vaccination with LmAIO reduces HCC tumor burden in mice and induces Th1 responses

**Fig. 4:**
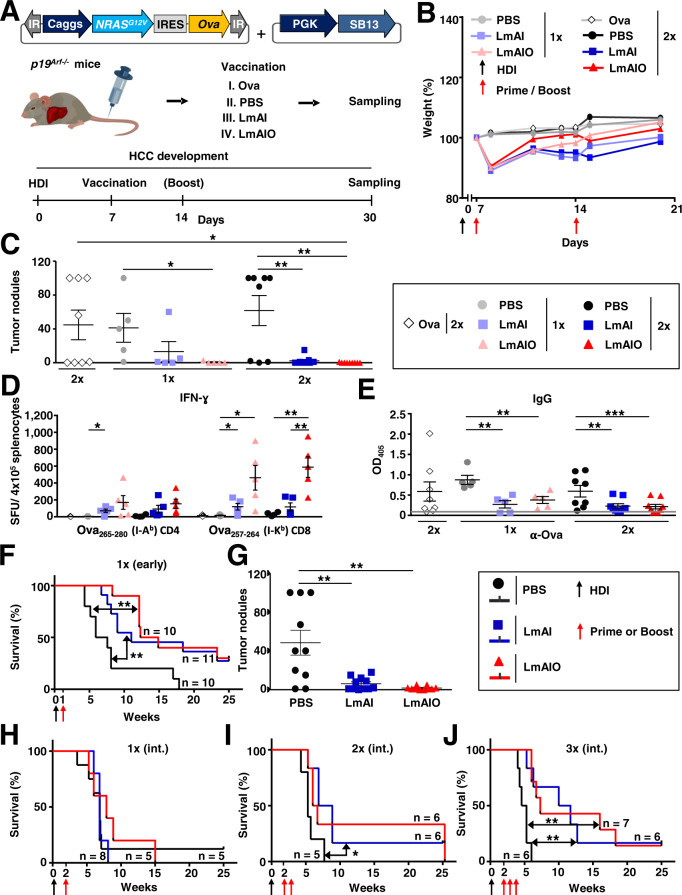
Therapeutic immunization with live-attenuated LmAIO induces strong Th1 responses and reduces HCC tumor burden. **A** Experimental setup to study the therapeutic potential of LmAIO and respective controls in HCC-Ova settings. **B** Weight development of mice after therapeutic vaccine administration. **C** Quantification of macroscopic HCC tumor nodules in all murine groups described in (**A**). **D** IFN-ɣ ELISPOT analysis on splenocytes re-stimulated with CD4- and CD8-specific Ova peptides. **E** ELISA to detect Ova-specific IgG in serum of vaccinated mice. **F**, **G** Kaplan–Meier survival curve (**F**) and intrahepatic tumor burden (**G**) of p19^Arf−/−^ mice vaccinated on day 7 after HDI (early HCC stage). **H**–**J**, Kaplan–Meier survival curves of p19^Arf−/−^ mice vaccinated once (**H**), twice (**I**), or three times (**J**), starting 14 days after HCC induction (int. = intermediate HCC stage). Numbers of animals per group in (**B**, **C**, **E**) single vaccination (PBS *n* = *5*, LmAI *n* = *5*, LmAIO *n* = *5*), two vaccinations (Ova *n* = *8*, PBS *n* = *8*, LmAI *n* = *9*, LmAIO *n* = *10*); in (**D**) single vaccination (PBS *n* = *5*, LmAI *n* = *5*, LmAIO *n* = *5*), two vaccinations (Ova *n* = *3*, PBS *n* = *4*, LmAI *n* = *5*, LmAIO *n* = *5*); in (**F**–**J**) numbers of animals are depicted in the plots. Data were analyzed using unpaired *t*-test and Mantel–Cox test for survival. *P < 0.05, **P < 0.01, ***P < 0.001. Shown are mean ± SEM. SFU, spot-forming units.

We further analyzed the therapeutic potential of LmAIO strain performing therapeutic vaccination in early HCC (day 7 after HDI, Supplementary Table S1). P19^Arf−/−^ mice were vaccinated once or twice after HCC-Ova induction and sampled 30 days after HDI (Fig. 4A). Primary therapeutic administration of Listeria resulted in a transient weight loss, while no obvious weight loss occurred upon secondary exposure to LmAI and LmAIO (Fig. 4B). Importantly, LmAIO vaccine was safe in HCC-bearing animals, as confirmed by histopathological examination of organs (Supplementary Table S2). Therapeutic vaccination with LmAIO and LmAI of HCC-bearing mice led to a strong reduction of tumor nodules in comparison to control groups, the effect was dose-dependent (Fig. 4C, Supplementary Fig. S4A).

Protection in treatment settings was associated with the induction of tumor/Ova-specific CD4 and CD8 IFN-ɣ responses (Fig. 4D). Although not significant, RAS^G12V^-specific IFN-ɣ responses were strongest in LmAIO-vaccinated animals (Supplementary Fig. S4B). No significant IL-4 responses towards Ova were detected (Supplementary Fig. S4C, D).

Strikingly, in line with reduced intrahepatic tumor burden, Ova-specific IgG levels were significantly reduced in both, LmAI- and LmAIO-vaccinated animals (Fig. 4E).

In conclusion, therapeutic vaccination of HCC-bearing mice with LmAIO induced strong IFN-Ɣ responses and reduced tumor burden, which correlated with decreased tumor-specific IgG.

### Therapeutic vaccination with LmAIO prolongs survival of mice harboring HCC

We next addressed whether therapeutic LmAIO vaccination would inhibit HCC-Ova development. We induced HCC-Ova via HDI and vaccinated animals at different HCC stages using different vaccination regimes. Single therapeutic vaccination with LmAIO at early HCC stage (day 7 after HDI) significantly delayed hepatocarcinogenesis (Fig. 4F). Tumor burden was significantly reduced in LmAI-vaccinated animals and only single tumors were observed in the LmAIO-vaccinated group. Aggressive multinodular HCC growth was present in PBS group (Fig. 4G).

Therapeutic vaccination with LmAIO at intermediate stage of HCC (day 14 after HDI) did not yield a significant survival benefit upon a single immunization (Fig. 4H). However, when animals were boosted once or twice, LmAIO as well as LmAI prolonged survival of mice (Fig. 4I-J). Intrahepatic tumor burden in Listeria-vaccinated groups decreased when compared to control independently of the applied vaccination regimen (Supplementary Fig. S4E).

Taken together, single therapeutic vaccination with LmAIO significantly prolonged the survival in early HCC, whereas at intermediate HCC stages the protection was dose-dependent and independent on the presence of tumor antigen in vaccine formulation.

### Prophylactic vaccination with LmAIO induces strong tumor-specific Th1 immunity, reduces tumor-specific IgG and prolongs survival in CCA

**Fig. 5:**
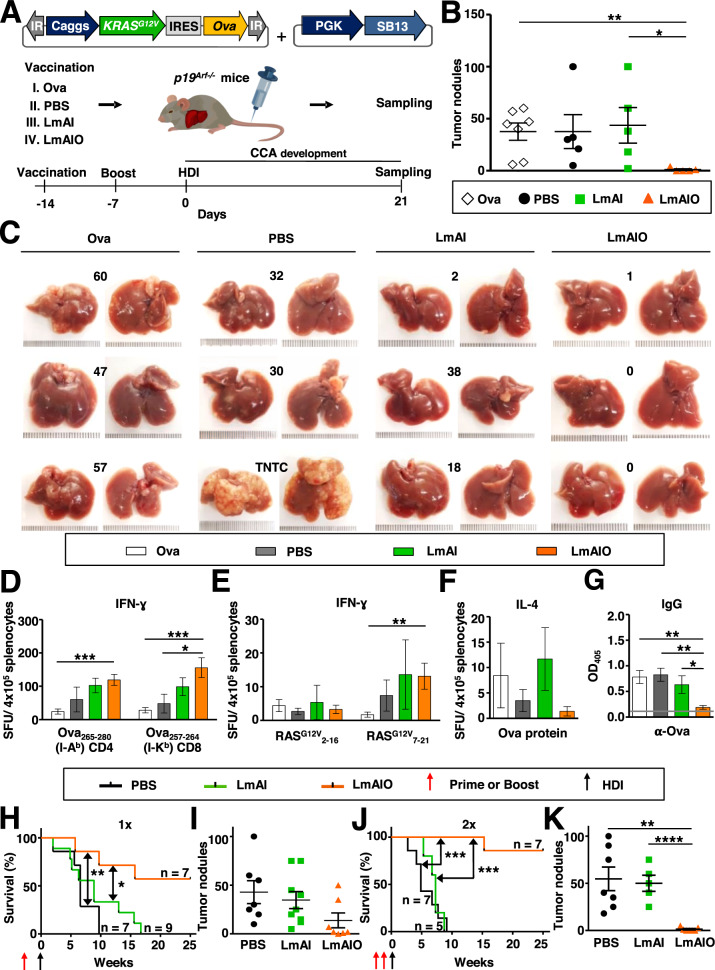
Prophylactic vaccination with live-attenuated LmAIO induces strong tumor-specific Th1 immune responses, a pronounced reduction of tumor-specific IgG, decrease in CCA tumor burden and prolonged survival of vaccinated animals. **A** Experimental setup to study the prophylactic potential of LmAIO in CCA-Ova settings. **B**, **C** Quantification of macroscopic CCA tumor nodules (**B**) and representative pictures of explanted livers at sampling with indicated tumor burden (**C**). TNTC, too numerous to count. **D**, **E** IFN-ɣ ELISPOT analysis on splenocytes re-stimulated with CD4 and CD8 Ova peptides (**D**) or oncogenic RAS^G12V^ peptides (**E**). **F** IL-4 ELISPOT analysis on splenocytes after re-stimulation with Ova protein. **G** ELISA to detect Ova-specific IgG in serum of vaccinated mice. **H**–**K**, Kaplan–Meier survival curves (**H**, **J**) and the corresponding intrahepatic tumor counts (**I**, **K**) detected in mice which received one (**H**, **I**) or two (**J**, **K**) doses of prophylactic vaccine. Numbers of animals per group in (**B**–**G**) (Ova *n* = *7*, PBS *n* = *5*, LmAI *n* = *5*, LmAIO *n* = *5*), in (**H**–**K**) numbers of animals are depicted in the plots. Data were analyzed using unpaired *t*-test and Mantel–Cox test for survival. *P < 0.05, **P < 0.01, ***P < 0.001, ****P < 0.0001. Shown are mean ± SEM. SFU, spot-forming units.

We next extended our investigations to another PLC subtype and tested the impact of prophylactic LmAIO vaccine in autochthonous CCA-Ova. P19^Arf−/−^ mice were vaccinated and boosted 14 and 7 days prior to the CCA-Ova induction (Fig. 5A). Due to the aggressive growth of CCA-Ova, animals were sampled on day 21 after HDI.

Prophylactic vaccination with Listeria caused a transient reduction of bodyweight (Supplementary Fig. S5A). LmAIO efficiently protected animals from CCA-Ova development in comparison to control groups (Fig. 5B, C). This protection correlated with the strong Ova-specific CD4 and CD8 IFN-ɣ responses (Fig. 5D). In addition, RAS^G12V^_7-21_-specific IFN-ɣ responses were shown significantly increased in LmAIO group (Fig. 5E). Interestingly, IFN-ɣ responses towards Listeria were less pronounced in LmAIO- compared to LmAI-vaccinated animals, similarly to the results in HCC-Ova (Supplementary Figs. S5C and S2C, respectively). Only minor Ova-specific IL-4 responses were detected in LmAIO-immunized mice (Fig. 5F, Supplementary Fig. S5B). Furthermore, LmAIO-vaccinated mice displayed significantly reduced Ova-specific IgG levels compared to all control groups (Fig. 5G), which strongly correlated with tumor burden results.

To determine long-term efficacy of prophylactic vaccination, we monitored survival in CCA-Ova settings. Single vaccination with LmAIO resulted in protection of 57% of mice for the observed time period with a prolongation of survival by 15 weeks, whereas LmAI alone resulted in a survival benefit of 7 weeks compared to PBS group (Fig. 5H). Administration of two LmAIO doses was sufficient to protect 86% of mice against CCA-Ova (Fig. 5J). Quantification of intrahepatic tumors revealed lower numbers in LmAIO-treated groups, which correlated with survival (Fig. 5I, K).

In summary, prophylactic vaccination with LmAIO induced strong Ova- and RAS^G12V^-specific Th1 responses, reduced tumor-specific IgG and protected from CCA development in a dose-dependent manner, similarly as shown for HCC.

### Therapeutic vaccination with LmAIO induces Th1 responses and prolongs survival of mice harboring CCA irrespective of the presence of tumor antigen in vaccine formulation

**Fig. 6:**
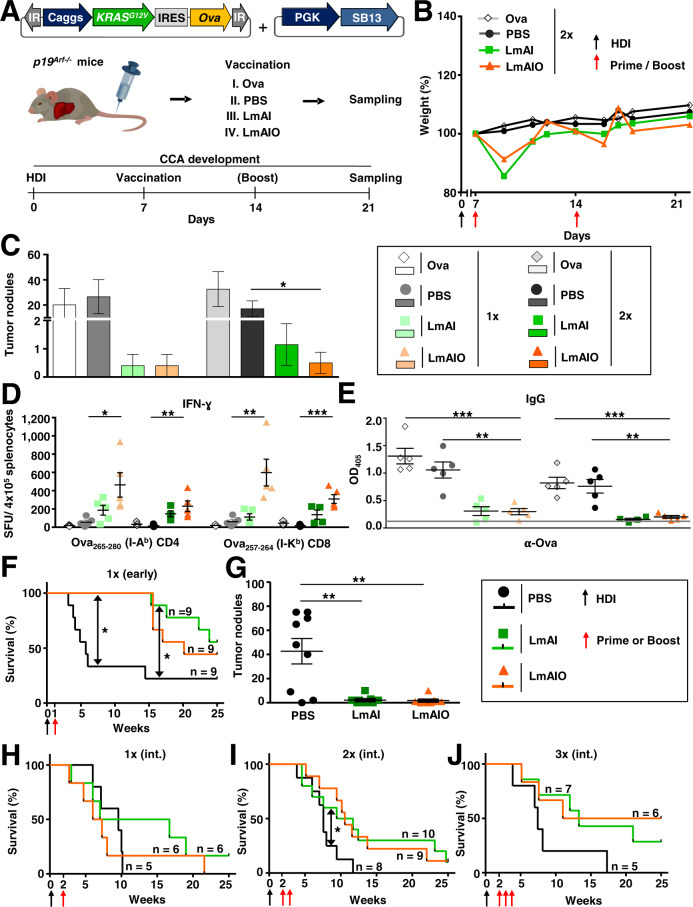
Therapeutic vaccination with live-attenuated LmAIO reduces CCA tumor burden, induces Th1 responses and prolongs survival in a dose-dependent manner. **A** Experimental setup to study the therapeutic potential of LmAIO in CCA-Ova settings. **B** Weight development after administration of the therapeutic vaccine. **C** Intrahepatic CCA nodule counts at sampling. **D** INF-ɣ ELISPOT analysis on splenocytes re-stimulated with the corresponding CD4 and CD8 Ova peptides. **E** ELISA to detect Ova-specific IgG in therapeutically vaccinated mice. **F**, **G** Kaplan–Meier survival curve of mice vaccinated on day 7 post-HDI (early CCA stage) (**F**) and corresponding tumor nodule counts (**G**) calculated in explanted livers. **H**–**J**, Kaplan–Meier survival curves of mice vaccinated 14 days post-HDI using one (**H**), two (**I**), or three (**J**) vaccinations (int. = intermediate CCA stage). Numbers of animals per group in (**B**–**E**) single vaccination (Ova *n* = *5*, PBS *n* = *5*, LmAI *n* = *5*, LmAIO *n* = *5*), two vaccinations (Ova *n* = *5*, PBS *n* = *5*, LmAI *n* = *4*, LmAIO *n* = *5*); in (**F**–**J**) numbers of animals are depicted in the plots. Data were analyzed using unpaired *t*-test and Mantel–Cox test for survival. *P < 0.05, **P < 0.01, ***P < 0.001. Shown are mean ± SEM. SFU, spot-forming units.

The impact of LmAIO on persistent CCA-Ova tumors was further examined in therapeutic vaccination studies. After CCA-Ova induction, mice were vaccinated either once or twice on day 7 and 14. On day 21 after CCA-Ova induction animals were sampled (Fig. 6A). We detected only a transient reduction of bodyweight in Listeria-vaccinated groups and no signs of pathological changes in analyzed organs (Fig. 6B, Supplementary Table S2).

Therapeutic vaccination with LmAI/LmAIO resulted in decreased tumor burden (Fig. 6C, Supplementary Fig. S6A), which correlated with induced Ova- and RAS^G12V^_7-21_-specific IFN-ɣ responses, whereas these were more pronounced in LmAIO group (Fig. 6D and Supplementary Fig. S6B, respectively). In contrast to the data in HCC-Ova, Ova-specific IL-4 responses were increased in LmAI and LmAIO compared to Ova and PBS groups in a dose-dependent manner in CCA-Ova (Supplementary Fig. S6C, D). The latter, however, did not influence Ova-specific IgG levels and they were decreased in LmAI/LmAIO groups (Fig. 6E), which in turn correlated with lower tumor counts (Fig. 6C).

To assess long-term therapeutic potential of LmAIO in CCA-Ova, survival was monitored. Vaccination at early CCA-Ova stage was independent on the presence of tumor model antigen in vaccine formulation. Both Listeria strains led to a significant protection against CCA (Fig. 6F, G). Therapeutic Listeria vaccination at the intermediate CCA stage was more efficient when three doses were administered and was not dependent on the presence of tumor model antigen (Fig. 6H-J). Tumor burden was decreased in LmAIO, compared to LmAI and PBS groups when two or three doses were administered (Supplementary Fig. S6E).

In conclusion, therapeutic vaccination with LmAIO decreased tumor burden, induced strong Ova- and RAS^G12V^-specific Th1 responses and reduced Ova-specific IgG, which correlated with the protection. Independently of the presence of tumor antigen, Listeria was capable to prolong the survival of CCA-Ova-bearing mice in a dose-dependent manner.

### Vaccination with LmAIO strongly reduces B lymphocyte counts in situ in HCC

**Fig. 7:**
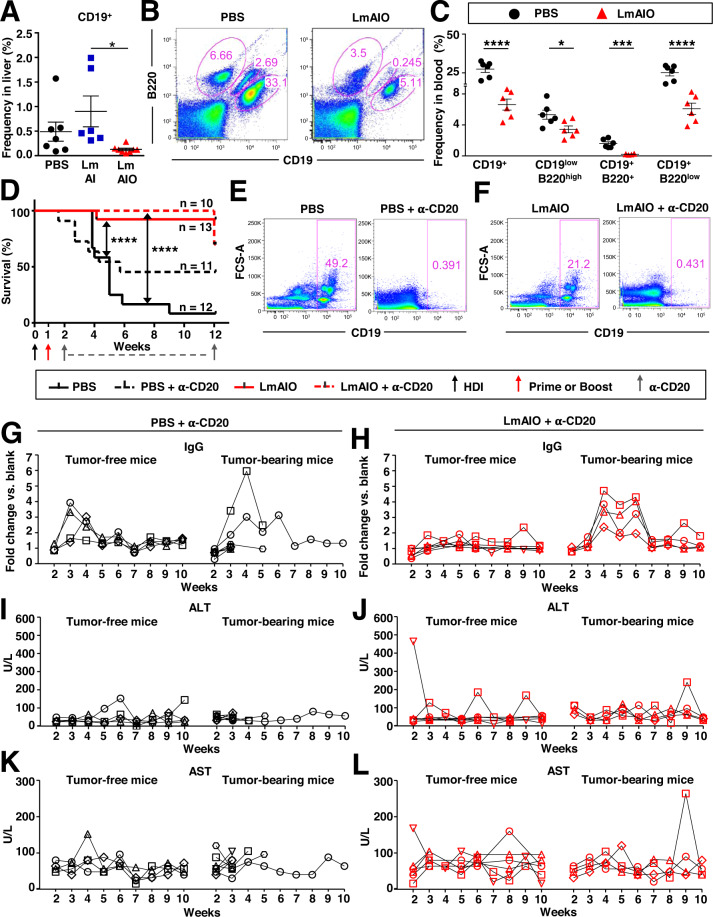
Vaccination with live-attenuated LmAIO strongly reduces frequencies of B lymphocytes in situ in HCC settings, whereas B cell-depletion does not yield any survival benefit. **A** Reduced frequencies of CD19^+^ B cells in livers of mice which received two prophylactic doses of LmAIO. **B**, **C** Reduced frequencies in different B cell populations (CD19^low^ B220^high^, CD19^+^ B220^+^, CD19^+^ B220^low^) in blood of mice 1 week after the therapeutic vaccination with LmAIO. **D** Kaplan–Meier survival curves of mice which received a combination therapy comprising a vaccination with LmAIO 7 days post-HDI and B cell-depletion therapy or respective controls. **E**, **F** Representative FACS plots on week 5 post-HDI of PBS- (**E**) and LmAIO-vaccinated (**F**) p19^Arf−/−^ animals with or without α-CD20 therapy, showing expression of CD19 on B cells in blood. **G**, **H** ELISA to detect Ova-specific IgG in plasma of PBS- (**G**) and LmAIO-vaccinated (**H**) animals which received α-CD20 therapy. Shown is kinetic for individual animals, separated into tumor-free and tumor-bearing counterparts. Plasma of mice was diluted 1:100 and used for IgG detection and thereafter for ALT/AST measurement shown in (**I–L**). **I**–**L** Kinetic of ALT (**I**–**J**) and AST (**K**–**L**) levels monitored in plasma of PBS- (**I**, **K**) and LmAIO-vaccinated (**J**, **L**) animals which received B cell-depletion therapy and separated into tumor-free and tumor-bearing counterparts. Numbers of animals per group in (**A**) (PBS *n* = *7*, LmAI *n* = *6*, LmAIO *n* = *8*), in (**C**) (PBS *n* = *6*, LmAIO *n* = *6*), in (**D**) (PBS *n* = *12*, LmAIO *n* = *13*, PBS + α-CD20 *n* = *11*, LmAIO+α-CD20 *n* = *10*), in (**G**, **I**, **K**) (tumor-free PBS + α-CD20 *n* = *4*, tumor-bearing PBS + α-CD20 *n* = *7*), in (**H**, **J**, **L**) (tumor-free LmAIO+α-CD20 *n* = *6*, tumor-bearing LmAIO+α-CD20 *n* = *4*). Data were analyzed using unpaired *t*-test. *P < 0.05, ***P < 0.001, ****P < 0.0001. Shown are mean ± SEM.

We further analyzed immune cell populations in liver and blood of vaccinated animals using flow cytometry (FACS). **In prophylactic settings** protection against HCC was accompanied by significantly reduced CD19^+^ B lymphocytes in livers of LmAIO-vaccinated mice (Fig. 7A, gating strategy shown in Supplementary Fig. S7A). Gating on CD19^+^MHCII^+^CD80^+^ B cells also showed a reduced activity of these cells in LmAIO-vaccinated group (Supplementary Fig. S7B). In line with B cells, two populations of dendritic cells (DC) and macrophages (MΦ), including their MHCII^+^CD80^+^ subpopulations, were decreased in livers of LmAIO group (Supplementary Fig. S7C-F and G-J, respectively).

Already 1 week after the early **therapeutic vaccination** of HCC-bearing animals, significant decreases within the B cell populations were detected in blood of LmAIO group in comparison to PBS controls (Fig. 7B, C, gating strategy performed on whole leukocytes is shown in Supplementary Fig. S7K). In particular, a highly significant four-fold-decrease in CD19^+^ as well as CD19^+^B220^low^ B cell subpopulations was detected in LmAIO-vaccinated animals in comparison to PBS group (Fig. 7C). Further, a decrease of CD19^+^ and CD19^+^MHCII^+^CD80^+^ B lymphocytes was detected locally in livers of mice, which received two therapeutic vaccinations with LmAIO (Supplementary Fig. S7L-N).

To further address the role of CD19^+^ B cells, we performed B cell-depletion therapy using α-CD20 neutralizing antibodies either alone or in combination with the LmAIO vaccination in HCC-Ova model (Fig. 7D-L). LmAIO vaccination was performed on day 7 post-HDI, B cell-depletion therapy was initiated as soon as the monitored Ova-specific IgGs were detected in plasma (Fig. 7D). Comparing PBS control and PBS + α-CD20 groups, we observed a survival benefit (8% and 45% survival, respectively), however, the differences between the groups were not significant (Fig. 7D). Interestingly, combination of LmAIO+α-CD20 did not demonstrate any significant survival benefit in comparison to LmAIO monotherapy (Fig. 7D). We performed kinetic studies and followed-up the IgG responses 10 weeks long in HCC mice which received either a B cell-depletion as monotherapy or a combination with LmAIO. Importantly, despite the efficient depletion of CD19^+^ B cells (Fig. 7E, F), Ova-specific IgG levels were still found upregulated and the highest IgG responses correlated with HCC development independent of therapeutic regime (Fig. 7G, H). Importantly, in contrast to α-CD20 monotherapy, the combination of LmAIO+α-CD20 was able to keep the IgG secretion on a constant low level in tumor-free mice (Fig. 7G, H). We did not observe any correlation between ALT/AST levels in plasma of mice and the IgG increase, α-CD20 therapy, or Listeria vaccination (Fig. 7I-L).

Taken together, the success of LmAIO-mediated protection against HCC is associated with a local reduction of activated DC and MΦ, local and systemic reduction of B cells and systemic reduction of tumor-promoting IgGs. B cell-depletion therapy combined with LmAIO did not demonstrate increased efficacy compared to LmAIO alone.

### Vaccination with LmAIO strongly decreases expression of inhibitory markers on CD4 and CD8 T cells in situ

**Fig. 8:**
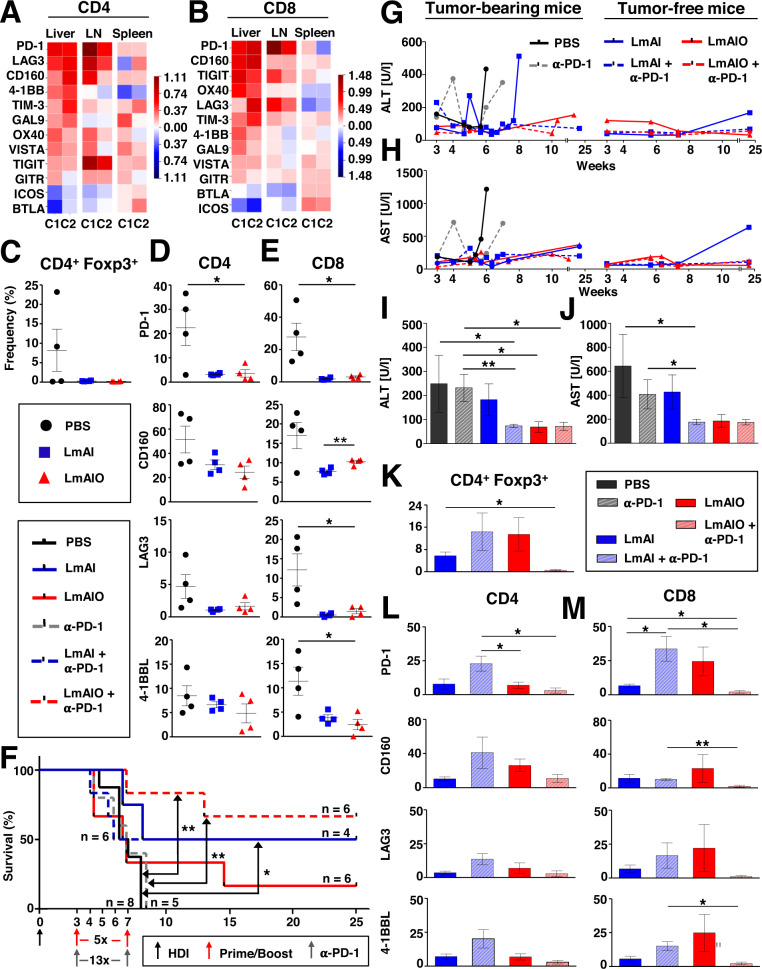
Therapeutic vaccination with live-attenuated LmAIO leads to reduced surface expression of ICI molecules on CD4/CD8 T lymphocytes and combination therapy with α-PD-1 blockade prolongs survival of mice with advanced HCC. **A**, **B** Microarray analysis on memory CD4 (**A**) and CD8 (**B**) T lymphocytes isolated from liver, LN and spleen of HCC-bearing mice (*NRAS*^*G12V*^*/c-Myc* genotype, *n* = *6*) versus two HCC-free controls (C1: *c-Myc, n* = *6*; C2: *NRAS*^*G12V*^*, n* = *6*). Gene expression values (log2-fold changes) detected in T lymphocytes of HCC-bearing mice compared to HCC-free controls are depicted in a heatmap with upregulated (> 0, marked in red) and downregulated gene expression (< 0, marked in blue). C, CD4^+^ Foxp3^+^ cells in livers of therapeutically vaccinated mice. **D**, **E** ICIs expression on CD4 (**D**) and CD8 (**E**) T cells in livers of therapeutically vaccinated animals. **F** Kaplan–Meier survival of HCC-Ova-bearing mice subjected to single and combination therapeutic regimes comprising LmAIO and α-PD-1 blockade, administered at advanced stages of the disease, at 3.5 weeks after HDI. **G**, **H** Kinetic of ALT (**G**) and AST (**H**) levels in plasma in mice which received combination therapy or respective controls. Shown are mean values per group, separated into tumor-bearing and tumor-free counterparts. **I**, **J** ALT (**I**) and AST (**J**) levels at sampling measured in plasma of mice which received combination therapy or respective controls. **K–M** Normalized data of tumor-bearing vs. tumor-free mice upon single and combination therapeutic regimes on CD4 and CD8 T cells in livers showing CD4^+^ Foxp3^+^ (**K**) and ICIs on CD4 (**L**) and on CD8 (**M**) T cells. Numbers of animals in (**C**–**E**) (PBS *n* = *4*, LmAI *n* = *4*, LmAIO *n* = *4*), numbers of animals in (**F**) are shown in the plot, in (**G**, **H**) tumor-bearing (PBS *n* = *4*, α-PD-1 *n* = *5*, LmAI *n* = *5*, LmAIO *n* = *4*, LmAI+α-PD-1 *n* = *8*, LmAIO+α-PD-1 *n* = *3*) tumor-free (LmAI *n* = *2*, LmAIO *n* = *1*, LmAI+α-PD-1 *n* = *2*, LmAIO+α-PD-1 *n* = *1*), in (**I**, **J**) (PBS *n* = *4*, α-PD-1 *n* = *5*, LmAI *n* = *7*, LmAIO *n* = *5*, LmAI+α-PD-1 *n* = *9*, LmAIO+α-PD-1 *n* = *4*), in (**K**–**M**) tumor-bearing (LmAI *n* = *4*, LmAIO *n* = *4*, LmAI+α-PD-1 *n* = *5*, LmAIO+α-PD-1 *n* = *3*), tumor-free (LmAI *n* = *2*, LmAIO *n* = *1*, LmAI+α-PD-1 *n* = *2*, LmAIO+α-PD-1 *n* = *1*). Data were analyzed using unpaired *t*-test and Mantel–Cox *t*est for survival. *P < 0.05, **P < 0.01. Shown are mean ± SEM.

We further investigated the expression of ICI molecules on CD4 and CD8 T cells in HCC-bearing animals. Unbiased microarray analyses on CD4 and CD8 T cells isolated from livers, lymph nodes (LN), and spleens of mice harboring aggressive HCCs (genotype *NRAS*^*G12V*^*/c-Myc*) showed a strong upregulation of several ICIs in situ in livers and LN of HCC-bearing animals in comparison to both tumor-free controls (C1 and C2). In CD4 T cells, we detected overexpression of PD-1, LAG3, CD160, 4-1BB and TIM-3, whereas PD-1, CD160, TIGIT, OX40 and LAG3 were overexpressed in CD8 T cells (Fig. 8A, B). Further comparison of ICIs on CD4 and CD8 T cells in livers of HCC-bearing and HCC-free mice independent on vaccination strategy revealed a significant upregulation of CD160, TIM-3, and PD-1 molecules on CD4 and CD8 T lymphocytes in HCC-Ova-harboring livers (Supplementary Fig. S8A, B, gating strategy shown in Supplementary Fig. S8C).

We further investigated whether live-attenuated Listeria had any impact on ICIs expression in therapeutically vaccinated animals. Importantly, already on day 7 after therapeutic vaccination with LmAIO in HCC we detected a significant reduction of PD-1, CD160, LAG3, and 4-1BBL on CD4/CD8 T cells in blood (Supplementary Fig. S8D-F). Furthermore, Foxp3^+^ T cell counts were reduced in livers of mice which received two doses of therapeutic vaccination of LmAI/LmAIO in comparison to PBS group (Fig. 8C). Therapeutic vaccination further led to a significant reduction of PD-1 expression and also dampened the expression of CD160, LAG3, and 4-1BBL on CD4/CD8 T cells in livers of LmAI- and LmAIO-vaccinated animals in comparison to control (Fig. 8D, E).

Taken together, LmAIO demonstrated a powerful capacity in reducing ICIs molecules on CD4/CD8 T lymphocytes in blood and livers of HCC-bearing animals.

### Combination therapy using live-attenuated LmAIO vaccine and α-PD-1 blockade prolongs survival at advanced HCC stage

Due to the highest overexpression in the PD-1 inhibitory molecule on CD4/CD8 T cells in HCC-bearing animals (Fig. 8A, B, Supplementary Fig. S8A, B), we performed a combination therapy comprising α-PD-1 blockade and vaccination with Listeria strains at a very advanced malignant liver disease stage (3.5 weeks after HCC-Ova induction, Supplementary Table S1). Remarkably, the combination of LmAIO+α-PD-1 therapy prolonged the survival of animals by 17 weeks in comparison to PBS-treated group and protected against HCC progression more efficiently than LmAI+α-PD-1 combination or LmAI and LmAIO monotherapy (Fig. 8F). Of note, 10 weeks after HCC-Ova induction all mice treated either with PBS or α-PD-1 single therapy, died. By week 12, mice with a combination of LmAIO+α-PD-1 therapy showed 83% survival in comparison to 33% LmAIO monotherapy and 50% LmAI monotherapy or LmAI+α-PD-1 combination therapy, respectively (Fig. 8F).

In contrast to all other groups tested, LmAIO as monotherapy or in combination with α-PD-1 was able to decrease ALT and AST levels in plasma of tumor-bearing and tumor-free mice during the entire survival kinetic study (Fig. 8G, H). A sampling (either due to tumor development or at 25 weeks post-HDI for all survivors), PBS-, α-PD-1- and LmAI-treated groups showed a significant increase of ALT/AST levels whereas LmAI+α-PD-1, LmAIO, and LmAIO+α-PD-1 therapeutic groups showed decreased ALT/AST (Fig. 8I, J).

Furthermore, Tregs (Fig. 8K) as well as ICIs on CD4 and CD8 T cells (Fig. 8L, M) were strongly reduced in livers of LmAIO+α-PD-1 group in comparison to otherwise treated animals. The latter data correlated with the observed survival (Fig. 8F).

In addition, we followed-up the ICIs in blood: tumor-free and tumor-bearing animals which received LmAIO+α-PD-1 therapy demonstrated a predominantly low level of ICIs, whereas all other groups demonstrated elevated levels of ICIs on CD4/CD8 T cells (Supplementary Fig. S8G, H).

Taken together, combination therapy LmAIO+α-PD-1 significantly rescued mice against advanced HCC-Ova progression while reducing Tregs and keeping liver inflammation and ICIs expression on CD4/CD8 T cells in blood and liver under constant control.

## Discussion

In this study, we demonstrated the safety and efficacy of LmAI-based vaccine strain in premalignant liver disease (fibrosis) and hepatobiliary cancers, using autochthonous murine models, which highly reflect human disease [11, 14, 19, 26–28]. This is the first report on the use of live-attenuated Listeria in fibrosis and CCA. Also, for the first time two autochthonous models of PLC (HCC and CCA) were compared side-by-side, demonstrating a high and comparable efficacy of Listeria-based vaccine.

Double-deleted vaccine strain LmAI has several important advantages: it can efficiently deliver large tumor antigens; it can be efficiently taken up by antigen-presenting cells, thereby inducing potent specific CD4/CD8 T cell responses due to the expression of important virulent factors like listeriolysin O [15, 16]; it is safe even in advanced cancers as previously reported [10, 15, 16] and also confirmed in our study. We further demonstrated that the strain did not induce additional liver injury in fibrosis or in early and advanced aggressive liver cancers. Our data are in line with the several clinical trials confirming a very efficient acceptance of the *i.v*. administered live-attenuated Listeria in patients with solid cancers [10, 29, 30]. Remarkably, early therapeutic vaccination with Listeria was also more efficacious than standard therapy sorafenib: LmAIO-vaccinated mice demonstrated a prolongation of survival by 48 days, whereas only 8 days survival benefit was induced by sorafenib in *NRAS*^*G12V*^-driven HCCs [26].

In this study, we thoroughly characterized a protective immune mechanism, which Listeria induced in prophylactic and therapeutic vaccination settings, thereby keeping the PLC development under control (the protective mechanism is summarized in a graphical abstract). The mechanism first relies on the strong induction of a Th1-polarized immune response, characterized by strong IFN-ɣ^+^ CD4/CD8 T cell responses towards the antigen delivered by Listeria (model antigen Ova), which fully correlate with several in vivo studies [31–33]. Our data further provide evidence that LmAIO strain is able to induce Th1 immune response against tumor antigens in HCC/CCA settings, thereby promoting breakdown of tolerance to self-antigens as previously reported [16] as well as epitope spreading [20, 21]. The latter resulted in IFN-ɣ response against oncogenic RAS^G12V^. IFN-ɣ responses towards endogenous oncogenes and generally epitope spreading mechanism [20, 21] are very important prerequisites for clinical vaccines based on Listeria, especially in unknown genotype of the tumor.

The protective mechanism induced by Listeria delivering tumor antigen further relies on a dramatic decrease of tumor antigen-specific IgGs. The latter correlated with reduced intrahepatic tumor burden and protection against PLC. Antibodies against Listeria were not involved in this mechanism. These observations are in line with the current notion that antibodies are not required for controlling Listeria infection [34]. In contrast, high titers of tumor antigen (Ova)-specific IgG antibodies were observed in tumor-bearing mice. These findings suggest a strong tumor-promoting role of tumor-specific IgGs, which could serve as biomarkers of PLC development. Consequently, tumor-bearing mice demonstrated a strong reduction of Th1 responses against tumor antigens. In line with our findings, a recent study in breast cancer reported that higher levels of ex vivo IgG responses to breast cancer antigens were associated with shorter survival and lower tumor-infiltrating CD8^+^ T cell counts [35]. Moreover, there are several reports describing a pro-tumorigenic role of cancer-derived IgGs, which were shown to induce inflammation, production of reactive oxygen species and tumor immune escape, thereby enhancing proliferation of cancer cells [36, 37].

Decrease of Ova-specific antibodies was also well in line with the local and systemic reduced frequency of CD19^+^ B cells in LmAIO-vaccinated animals. Tumor-promoting role of B cells was recently reported by M. Karin laboratory in non-alcoholic fatty liver disease [38]. Moreover, Gu et al., recently reported that B cells play a pro-tumorigenic role and can selectively promote breast cancer metastasis by HSPA4-targeting IgG [39]. Decrease of CD19^+^ B cells upon vaccination with live-attenuated Listeria in PLC is demonstrated for the first time in our study. Surprisingly, an additional depletion of B cells neither resulted in any significant survival benefit nor controlled tumor-specific IgG levels. Our data support the finding of DiLillo et al., showing that α-CD20 therapy does not reduce IgG-producing plasma B cells [40], therefore a more specific targeting of plasma B cell population could be of advantage and needs to be investigated in follow-up studies.

Our results further demonstrate that live-attenuated Listeria led to the decrease of DCs and MΦ, which also seem to be involved in the tumor-promoting mechanism, as we detected subpopulations of these cells to be reduced locally in livers upon vaccination with Listeria and upregulated in mice with tumors. Our data are in line with a hypothesis of R. Schwabe postulating, that “HCC is a never healing wound” [41], suggesting that Listeria vaccine mediates reduction of local inflammation in liver, thereby strongly decreasing activated DC/MΦ in liver. Moreover, in α-PD-1 experiments, we observed that the successful combination therapy LmAIO+α-PD-1 also resulted in the lowest ALT/AST plasma levels determined in kinetic studies, suggesting that live-attenuated LmAIO vaccine kept liver inflammation under constant control. The detailed mechanism why Listeria is able to decrease tumor-specific IgGs, B cell, DC, MΦ counts and ALT/AST in liver cancer remains to be elucidated.

We showed for the first time, that live-attenuated Listeria vaccine reduced the expression of several important tumor-promoting ICIs on CD4/CD8 T lymphocytes. Listeria also strongly downregulated Tregs counts, similarly to previous reports [31, 42]. Decrease of ICIs was observed in early and late therapeutic vaccination and correlated with protection against HCC.

The combination therapy comprising vaccination with Listeria delivering tumor antigen and a blockade of the most abundant ICI, PD-1, showed a great perspective even at advanced cancer stage and efficiently prolonged survival of HCC-bearing animals, whereas single therapies were not successful. The latter findings once again supported our conclusions, that only a combination therapy will be efficient in advanced HCC/CCA and needs to be evaluated in clinical trials and subsequently implemented in clinic [10, 43]. Efficacy of a combination therapy using Listeria has been recently shown in transplantable HCC model [44] and in LmAI-based Annexin A2-targeting cancer immunotherapy in pancreatic cancer models [45]. Importantly, Xu et al. showed that Listeria-based vaccine Lmdd-MPFG plays a role of a tumor microenvironmental modifier that resides the tumor-associated MΦ, promotes M1 MΦ polarization and generates a tumor microenvironment friendly to α-PD-1 therapy, thereby facilitating success of the combination therapy [44].

Taking into account the upregulation of several ICI parameters on T cells in tumor-bearing animals, we do not exclude that a combination comprising Listeria vaccine and a blockade of CD160, 4-1BBL and/or a combination thereof will be also efficient. Such hypothesis is also supported by Gilley and Dube who successfully combined anti-CTLA4 or anti-PD-L1 blockade with Listeria expressing Ova in melanoma [46].

In recent years, several researchers/clinicians proposed to apply prophylactic vaccination in patients who suffer from chronic liver disease and are therefore at risk of cancer development. Especially now, in a new era of delivery platform vaccines emerged due to COVID-19 pandemics, live-attenuated Listeria can be implemented as a safe and very potent delivery platform of tumor antigens and inducer of strong anti-cancerous immune responses in patients who are at risk of cancer. Importantly, despite the developed liver fibrosis disease (Ishak score F3), LmAIO induced strong Th1 responses towards the delivered tumor model antigen. In addition, our results on prophylactic vaccination followed by a subsequent challenge with HCC/CCA confirmed a strong protection against both PLC types and underline the importance of future implementation of preventive vaccination regimes against HCC/CCA at least in risk groups (patients with advanced fibrosis, cirrhosis, and chronic cholangitis). Although, a thorough characterization and toxicity studies have to be still performed using advanced preclinical models of cirrhosis and cholangitis, the data obtained by us in liver fibrosis look very encouraging. For the establishment of prophylactic vaccines, most abundant tumor-associated antigens have to be defined using available cancer registry [47, 48].

## Conclusion

Our study demonstrated live-attenuated LmAI vaccine strain as safe and efficient delivery platform of liver cancer antigens and potent inducer of protective immune mechanism which enables prevention and/or abrogation of aggressive hepatobiliary cancers.

## Supplementary information

Supplementary Information

Supplementary Figures
