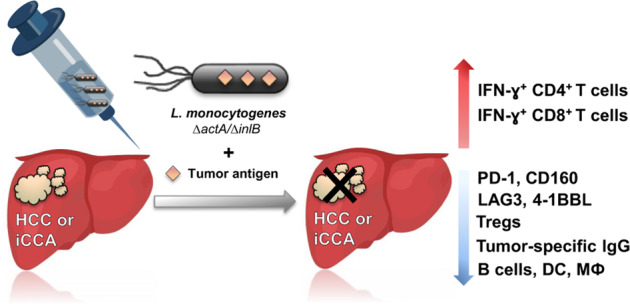# Correction: Safety and efficacy of prophylactic and therapeutic vaccine based on live-attenuated *Listeria monocytogenes* in hepatobiliary cancers

**DOI:** 10.1038/s41388-022-02261-6

**Published:** 2022-04-13

**Authors:** Inga Hochnadel, Lisa Hoenicke, Nataliia Petriv, Lavinia Neubert, Elena Reinhard, Tatjana Hirsch, Juan Carlos Lopez Alfonso, Huizhen Suo, Thomas Longerich, Robert Geffers, Ralf Lichtinghagen, Carlos Alberto Guzmán, Heiner Wedemeyer, Henrike Lenzen, Michael Peter Manns, Dunja Bruder, Tetyana Yevsa

**Affiliations:** 1 Department of Gastroenterology, Hepatology and Endocrinology, Hannover Medical School (MHH), Hannover, Germany; 2 Institute of Pathology, MHH, Hannover, Germany; 3 Department of Vaccinology and Applied Microbiology, Helmholtz Centre for Infection Research (HZI), Braunschweig, Germany; 4 Immune Regulation Group, HZI, Braunschweig, Germany and Infection Immunology Group, Institute of Medical Microbiology and Hospital Hygiene, Otto-von-Guericke University Magdeburg, Magdeburg, Germany; 5 Department of Systems Immunology, Technical University Braunschweig and HZI, Braunschweig, Germany; 6 Institute of Pathology, Heidelberg University Hospital, Heidelberg, Germany; 7 Genome Analytics, HZI, Braunschweig, Germany; 8 Department of Clinical Chemistry, MHH, Hannover, Germany

**Keywords:** Liver cancer, Immunotherapy

Correction to: *Oncogene* 10.1038/s41388-022-02222-z, published online 16 February 2022

The graphic abstract was missing from this article and should have appeared as below.

Protective immune mechanism induced by live-attenuated double-deleted *L. monocytogenes ∆actA/∆inlB* vaccine strain delivering tumor antigens keeps hepatobiliary malignancies under control. Live-attenuated, double-deleted *L. monocytogenes ∆actA/∆inlB* strain expressing model tumor antigen was used in both, prophylactic and therapeutic vaccination settings. Vaccination was safe and led to: (i) induction of protective tumor-specific Th1 immune responses in premalignant and malignant stages and strong increase of tumor-specific IFN-ɣ^+^ CD4 and CD8 T cells; (ii) decrease of T regulatory cells; (iii) downregulation of several tumor-promoting ICI molecules (PD-1, CD160, LAG3, 4-1BBL) on CD4/CD8 T lymphocytes; (iv) decrease of tumor-specific IgG in serum, and (v) decrease of B lymphocytes, DC and MΦ locally in livers. iCCA, intrahepatic cholangiocarcinoma.