# Supplementary material for: Safety and efficacy of prophylactic and therapeutic vaccine based on live-attenuated Listeria monocytogenes in hepatobiliary cancers
Source: Oncogene. 2022 Feb 16;41(14):2039–53. doi: 10.1038/s41388-022-02222-z (PMC8853207; doi:10.1038/s41388-022-02222-z)
Supplement: Supplementary file 1 — Supplementary Information [file 41388_2022_2222_MOESM1_ESM.docx]

**Supplementary Information**

**Abbreviations**

ActA actin assembly-inducing protein

ALT alanine aminotransferase

AST aspartate aminotransferase

BHI brain heart infusion

BSA bovine serum albumin

CCA cholangiocarcinoma

CCl_4_ tetrachloromethane

CFU colony-forming units

CK7 cytokeratin 7

DC dendritic cells

FACS flow cytometry

FDA Food and Drug Administration

GEO Gene Expression Omnibus

HCC hepatocellular carcinoma

HDI hydrodynamic tail vein injection

H&E hematoxylin and eosin

HZI Helmholtz Centre for Infection Research

iCCA intrahepatic cholangiocarcinoma

ICI immune checkpoint inhibitor

IFN-ɣ interferon-ɣ

IL interleukin

InlB internalin B

*i.p.* intraperitoneal

IR inverted repeats

IRES internal ribosome entry site

*i.v.* intravenous

LmAI *Listeria monocytogenes ∆actA/∆inlB*

LmAIO *Listeria monocytogenes ∆actA/∆inlB + Ova*

LN lymph node

MΦ macrophages

MHH Hannover Medical School

Ova Ovalbumin

PD-L1 programmed death-ligand 1

PFA paraformaldehyde

PGK phosphoglycerate kinase promoter

PLC primary liver cancer

RFP red fluorescent protein

SB13 Sleeping Beauty 13

SFU spot-forming units

SEM standard error of the mean

Th1 T helper 1

TNTC too numerous to count

Treg T regulatory cells

WT wild type

**Supplementary Tables**

**Supplementary Table S1. Kinetic of HCC and CCA development – early tumor development is observed already on day 7 after HDI.** *NRAS^G12V^-Ova* (for HCC-Ova) or K*RAS^G12V^-Ova* (for CCA-Ova) transposon constructs were co-delivered with transposase SB13 into p19^Arf-/-^ mice using HDI. Mice were sacrificed at different time points: days 7, 14, 21 and 30-34 after HDI. Livers were explanted and different (immuno)histochemical stainings (hematoxylin and eosin (H&E), Elastica van Giesson (reticulin staining), arginase 1 and CK7) of the tissue were performed and evaluated by experienced pathologists.

| **Days after HDI** | | | | |
| --- | --- | --- | --- | --- |
| **Cancer**  **Type /**  **Oncogene** | **7** | **14** | **21** | **30-34** |
| **HCC /**  ***NRAS^G12V^-Ova*** | Multiple neoplastic foci composed of highly atypical / pleomorphic cells, sometimes forming initial sarcomatoid carcinoma nodules. | Multiple  neoplastic foci composed of highly atypical / pleomorphic cells besides sarcomatoid carcinoma nodules. | Multiple sarcomatoid carcinoma nodules, sometimes with areas of ductal differentiation. | Poorly differentiated carcinoma (arginase^+^, loss of reticulin fibres in ilver staining) with focal necrosis, variable growth pattern: compact, focal trabecular, focal sarcomatoid; sometimes combined HCC-CCA with areas of ductal differentiation (CK7^+^).  **Conclusion:**  Histopathology consistent with poorly differentiated / dedifferentiated HCC, occasionally HCC-CCA. |
| **CCA /**  ***KRAS^G12V^-Ova*** | Multiple neoplastic foci composed of highly atypical / pleomorphic cells, sometimes forming initial sarcomatoid carcinoma nodules. | Several atypical micronodular lesions consistent with sarcomatoid carcinoma nodules, few with evidence of ductal differentiation. | Multiple sarcomatoid carcinomas, few with evidence of ductal differentiation | Moderately / poorly differentiated, predominantly glandular growing carcinoma (arginase^-^, CK7^+^) with focal necrosis and areas of sarcomatoid dedifferentiation.  **Conclusion:**  Histopathology consistent with moderately / poorly differentiated CCA. |

**Supplementary Table S2. Safety assessment of live-attenuated Listeria vaccine strain revealed no pathological changes in analyzed organs associated with vaccination, as confirmed using kinetic studies in HCC- and CCA-bearing animals.** *NRAS^G12V^-Ova* (for HCC-Ova) or *KRAS^G12V^-Ova* (for CCA-Ova) transposon constructs were co-delivered with transposase SB13 into livers of p19^Arf-/-^ mice via HDI. Mice were vaccinated with LmAIO strain on day 7 after HDI. Control group was treated with PBS. On day 14, 21 and 28 after HDI (according to day 7, 14 and 21 after vaccination) mice were sacrificed and different organs (liver, pancreas, spleen and heart) were isolated. Histopathologic examinations in tissue samples were performed by experienced pathologists. No significant histomorphological changes in association with vaccine administration in analyzed organs were detected. Only data for liver is shown.

| **14 days after HDI** | | |
| --- | --- | --- |
| **7 days after vaccination** | | |
| **Cancer type /**  **Oncogene** | **Vaccine** | **Liver** |
| **HCC /**  ***NRAS^G12V^-Ova*** | Lm AIO | Focal single cell necrosis with minimal infiltration of mononuclear cells.  No tumor (microscopic examination). |
|  | Control (PBS) | Focal single cell necrosis with minimal infiltration of mononuclear cells.  Initial tumor (microscopic examination). |
| **CCA /**  ***KRAS^G12V^-Ova*** | Lm AIO | Focal single cell necrosis with minimal infiltration of mononuclear cells.  No tumor (microscopic examination). |
|  | Control (PBS) | Focal mild portal lymphocytic aggregates and focal giant cell transformation, focal single cell necrosis with mild infiltration of mononuclear cells.  Initial tumor (microscopic examination). |
| **21 days after HDI** | | |
| **14 days after vaccination** | | |
| **Cancer type /**  **Oncogene** | **Vaccine** | **Liver** |
| **HCC /**  ***NRAS^G12V^-Ova*** | Lm AIO | Focal single cell necrosis with minimal infiltration of mononuclear cells.  Tumor (microscopic examination). |
|  | Control (PBS) | Focal single cell necrosis with minimal infiltration of mononuclear cells.  Tumor (microscopic examination). |
| **CCA /**  ***KRAS^G12V^-Ova*** | Lm AIO | Focal single cell necrosis with minimal infiltration of mononuclear cells.  Tumor (microscopic examination). |
|  | Control (PBS) | Focal single cell necrosis with minimal infiltration of mononuclear cells.  Tumor (microscopic examination). |
| **28 days after HDI** | | |
| **21 days after vaccination** | | |
| **Cancer type /**  **Oncogene** | **Vaccine** | **Liver** |
| **HCC /**  ***NRAS^G12V^-Ova*** | Lm AIO | Focal single cell necrosis with minimal infiltration of mononuclear cells.  Tumor (microscopic examination). |
|  | Control (PBS) | Focal single cell necrosis with minimal infiltration of mononuclear cells. Central venules surrounded by mild lymphocytic aggregates.  Tumor (microscopic examination). |
| **CCA /**  ***KRAS^G12V^-Ova*** | Lm AIO | Focal single cell necrosis with minimal infiltration of mononuclear cells.  Tumor (microscopic examination). |
|  | Control (PBS) | Focal single cell necrosis with minimal infiltration of mononuclear cells.    Tumor (microscopic examination). |

**Supplementary Figure Legends**

**Supplementary Fig. S1. Vaccination with live-attenuated LmAIO does not elicit histopathological changes in liver, heart, pancreas and spleen. A,** 24 hours after vaccination with 1 x 10^7^ CFU of LmAI or LmAIO, mice were sampled and organs were analyzed for bacterial burden. The average CFU/organ, or CFU/0.5 ml for blood, pooled from all experiments (*n=20*) is plotted. **B-E,** H&E stainings of explanted liver (**B**), heart (**C**), pancreas (**D**) and spleen (**E**) of mice 30 days after vaccination with 0.1 LD_50_ LmAIO shows no histopathological changes. Magnification x 100 (**B, C, E**), x 200 (**D**). **F-H,** H&E staining of liver 30 days after 0.1 LD_50_ vaccination showing liver parenchyma with focal accumulations of pigmented macrophages. Magnification x 100 (**F**), x 200 (**G**), x 400 (**H**). **I-J,** Weight development of p19^Arf-/-^ (**I**) and C57BL/6J WT (**J**) mice, respectively, after prophylactic vaccinations with LmAIO, LmAI or control treatment with endotoxin-free Ova protein and PBS, administered on days -14 and -7. Numbers of animals per group are depicted in the legend of Fig. 2 and Supplementary Figure 2. Data were analyzed using unpaired *t*-test. ****P* < 0.001, *****P* < 0.0001. Shown are mean ± SEM. CFU, colony-forming units.

**Supplementary Fig. S2. Prophylactic immunization with live-attenuated LmAIO reduces tumor burden and induces comparable immune responses in p19^Arf-/-^ and WT mice.** WT and p19^Arf-/-^ mice received prophylactic vaccinations and immune responses were analyzed. **A, B,** IFN-ɣ ELISPOT analysis on WT splenocytes re-stimulated with either the corresponding CD4 and CD8 Ova peptides (**A**) or oncogenic RAS^G12V^ peptides (**B**). **C,** IFN-ɣ ELISPOT analysis on splenocytes from p19^Arf-/-^ and WT mice re-stimulated with LmAI or LmAIO whole cell antigen. **D,** IL-4 ELISPOT analysis on WT splenocytes re-stimulated with LPS-free Ova protein. **E, F,** IL-4 ELISPOT analysis on splenocytes from p19^Arf-/-^ and WT mice re-stimulated with CD4 Ova peptide (**E**) or oncogenic RAS^G12V^ peptides (**F**). **G-J,** ELISA to detect IgM (**G, H**) and IgG (**I, J**) antibodies against LmAI and LmAIO whole cell antigen in serum of p19^Arf-/-^ and WT mice after prophylactic vaccination and subsequent intrahepatic overexpression of *NRAS^G12V^-Ova*. Grey line in ELISA represents blank values. Numbers of animals for p19^Arf-/-^ mice are depicted in the legend of Fig. 2. Numbers for WT mice (Ova *n=16*, PBS *n=16*, LmAI *n=16*, LmAIO *n=19*). Data were analyzed using unpaired *t*-test. **P* < 0.05, ***P* < 0.01, ****P* < 0.001, *****P* < 0.0001. Shown are mean ± SEM. SFU, spot-forming units.

**Supplementary Fig. S3. Live-attenuated LmAIO vaccine is a potent inducer of Th1 responses in mice with fibrosis. A,** IFN-ɣ ELISPOT analysis on splenocytes re-stimulated with LmAI or LmAIO whole cell antigen. **B-E,** ELISA on LmAI-specific IgM (**B**) and IgG (**C**) and LmAIO-specific IgM (**D**) and IgG (**E**). Numbers of animals per group are depicted in the legend of Fig. 3. Data were analyzed using unpaired *t*-test. **P* < 0.05, ***P* < 0.01, ****P* < 0.001, *****P* < 0.0001. Shown are mean ± SEM. SFU, spot-forming units.

**Supplementary Fig. S4. Early therapeutic vaccination with live-attenuated LmAIO reduces HCC tumor burden in mice. A,** Representative photographs of livers at sampling of mice vaccinated once (1x) or twice (2x) with LmAI, LmAIO or respective controls. TNTC, too numerous to count. **B,** IFN-ɣ ELISPOT analysis on splenocytes of p19^Arf-/-^ mice after re-stimulation with oncogenic RAS^G12V^ peptides. **C, D,** IL-4 ELISPOT analysis of splenocytes from p19^Arf-/-^ mice after re-stimulation with LPS-free Ova protein (**C**) and CD4 Ova peptide (**D**). **E,** Quantification of HCC tumor nodules in mice that received one, two or three vaccination doses of LmAI, LmAIO, or PBS as control, respectively, beginning on day 14 after HCC induction. Numbers of animals per group are depicted in the legend of Fig. 4. Data were analyzed using unpaired *t*-test. ***P* < 0.01. Shown are mean ± SEM. SFU, spot-forming units.

**Supplementary Fig. S5. Prophylactic vaccination with LmAIO and LmAI leads to moderate changes in bodyweight and induction of Th1 responses in CCA settings. A,** Weight development of p19^Arf-/-^ mice vaccinated with LmAI, LmAIO or Ova protein and PBS as controls 14 and 7 days prior to CCA induction. **B,** IL-4 ELISPOT analysis on splenocytes of p19^Arf-/-^ mice re-stimulated with CD4 Ova peptide. **C,** ELISPOT analysis on splenocytes of p19^Arf-/-^ mice re-stimulated with LmAI and LmAIO whole cell antigen to detect IFN-ɣ. Numbers of animals per group are depicted in the legend of Fig. 5. Data were analyzed using unpaired *t*-test. ****P* < 0.001. Shown are mean ± SEM. SFU, spot-forming units.

**Supplementary Fig. S6. Therapeutic vaccination of CCA-Ova-bearing mice leads to decrease in intrahepatic tumor counts.** Mice were vaccinated once (1x) or twice (2x) and sampled as described in Fig. 6A. **A,** Representative pictures of explanted mouse livers 21 days after HDI. **B,** IFN-ɣ ELISPOT analysis on splenocytes of p19^Arf-/-^ mice after re-stimulation with oncogenic RAS^G12V^ peptides. **C, D,** IL-4 ELISPOT analysis on splenocytes of p19^Arf-/-^ mice after re-stimulation with LPS-free Ova protein (**C**) and CD4 Ova peptide (**D**). **E,** Tumor counts in explanted mouse livers receiving one, two or three vaccination doses on day 14 after CCA-Ova induction. Numbers of animals per group are depicted in the legend of Fig. 6. Data were analyzed using unpaired *t*-test. **P* < 0.05, ***P* < 0.01. Shown are mean ± SEM. SFU, spot-forming units.

**Supplementary Fig. S7. Therapeutic vaccination with live-attenuated LmAIO reduces B cells, DC and MΦ in HCC-Ova. A,** Gating strategy to define B lymphocytes in livers of HCC-Ova-harboring mice. **B-J,** Reduced frequencies in different populations of B cells (**B**), two populations of DC (CD11c^+^ CD11b^+^ and CD11c^+^ CD11b^-^) (**C-F**) and two populations of MΦ (F4/80^+^ CD11b^+^ and F4/80^+^ CD11b^-^) (**G-J**) in livers of mice which received two prophylactic doses of LmAIO. **K**, Gating strategy to define various B cells populations (CD19^low^ B220^high^, CD19^+^ B220^+^, CD19^+^ B220^low^) in blood of mice which received a vaccination with LmAIO or PBS 7 days post-HDI. **L-N,** Frequencies of CD19^+^ B cells (**L**) as well as representative FACS plots (**M**) and diagram showing frequencies of CD19^+^ MHCII^+^ CD80^+^ B cells (**N**) in livers of mice, which received two therapeutic vaccinations. Numbers of animals per group in (**B-J**) (PBS *n=7*, LmAI *n=6*, LmAIO *n=8*), in (**L-N**) (PBS *n=4*, LmAI *n=4*, LmAIO *n=4*). Data were analyzed using unpaired *t*-test. **P* < 0.05, ***P* < 0.01, ****P* < 0.001. Shown are mean ± SEM.

**Supplementary Fig. S8. Therapeutic vaccination with live-attenuated Listeria is reducing the expression of ICI molecules on CD4 and CD8 T cells in blood and liver in HCC-bearing mice. A, B,** ICIs expression on CD4 and CD8 T cells in livers of tumor-bearing and tumor-free mice irrespective of vaccination strategy. **C, D,** Gating strategy for FACS analysis to define ICI molecules on CD4 and CD8 T cells in liver (**C**) and blood (**D**). **E, F,** ICIs expression on CD4 (**E**) and CD8 T lymphocytes (**F**) in blood 7 days after therapeutic vaccination. **G, H,** ICIs kinetic in blood on CD4 (**G**) and CD8 (**H**) T cells in mice administered with LmAIO+α-PD-1 combination therapy or respective controls in advanced HCC. Numbers of mice in (**A, B**) (tumor-bearing *n=6*, tumor-free *n=11*), in (**E, F**) (PBS *n=6*, LmAIO *n=6*), in (**G, H**) (tumor-bearing: PBS *n=4*, α-PD-1 *n=5*, LmAI *n=5*, LmAI+α-PD-1 *n=7*, LmAIO *n=4*, LmAIO+α-PD-1 *n=2*; tumor-free: LmAI *n=2*, LmAI+α-PD-1 *n=2*, LmAIO *n=1*, LmAIO+α-PD-1 *n=1*). Data were analyzed using unpaired *t*-test. **P* < 0.05, ***P* < 0.01, ****P* < 0.001, *****P* < 0.0001. Shown are mean ± SEM.

**Supplementary Materials and Methods**

***Animal experiments***

All animals (mice) used in this study as well as age and gender of animals are listed in Supplementary Table S3. All animal experiments and procedures were performed in compliance with ethical regulations and the approval of the Lower Saxonian State Office for Consumer Protection and Food Safety (LAVES, Niedersächsisches Landesamt für Verbraucherschutz und Lebensmittelsicherheit; AZ 18/2808, 15/1766, 13/1342, 15/1800). C57BL/6J mice were obtained from Charles River Laboratories. P19^Arf-/-^ mice [1] were obtained in a C57BL/6J background as described previously [2-6]. C57BL/6-Foxp3^tm1Flv^/J mice [7] were obtained from Jackson Laboratory (USA). All *in vivo* experiments were initiated in mice with 4-8 weeks of age. All animals were maintained under specific pathogen-free conditions in accordance with the institutional guidelines of the MHH/HZI (Germany). Food (standard diet) and water were provided to the animals *ad libitum*.

***Vector design***

*SB13* transposase, *NRAS^G12V^* (CaN), *NRAS^G12V^-IRES-Ova* (CaNIO) and *Myc-IRES-GFP* (CaMIG) encoding transposon vectors have been described previously [3, 5, 6, 8, 9]. CaN was used to generate an empty pCaggs transposon vector without gene of interest by replacing *NRAS^G12V^* by a multiple cloning site (pCaggs-MCS). The constitutively activated human *KRAS^G12V^* construct was provided by Dr. Engin Gürlevik, MHH, Germany, and was described previously [10, 11]. To generae the *KRAS^G12V^-IRES-Ova* transposon (CaKIO), *KRAS^G12V^* was PCR amplified and MluI and NotI restriction sites were attached using primers KRAS MluI fw (5’-ggaggaacgcgtatgactgagtataaacttg-3’) and KRAS NotI rv (5’-tgtagcggccgcgtattcacataactgtacacc-3’). This was inserted into pCaggs-MCS using AscI and NotI restriction sites to form CaK. *IRES-Ova* was PCR-amplified from CaNIO with primers IRES NotI fw (5’-ggaggagcggccgcatcgaggttaacgaattccgccc-3’ (NotI overang)) and Ova AgeI rv (5’-tcgccaaccggtttaaggggaaacacatctgc-3’ (AgeI overhang)) and inserted into CaK using the NotI and AgeI restriction sites resulting in CaKIO. All vectors and DNA primer sequences used in the study are listed in Supplementary Tables S4 and S5.

***Induction of autochthonous HCC and CCA***

To induce HCC-Ova and CCA-Ova development, we stably delivered transposable elements co-encoding *NRAS^G12V^-Ova* (CaNIO, see “Vector design” section) or *KRAS^G12V^-Ova* (CaKIO, see “Vector design” section), in combination with SB13 transposase into hepatocytes of p19^Arf-/-^ mice via HDI. To induce HCC (genotype *Nras^G12V^*/*c-Myc*) development, we stably delivered transposable elements encoding *Nras^G12V^* and *c-Myc* together with SB13 into hepatocytes of C57BL/6-Foxp3^tm1Flv^/J mice. Vectors (transposon and SB13 transposase) for HDI injection were prepared using QIAGEN EndoFree Maxi Kit (QIAGEN, Hilden, Germany). Transposon and transposase vectors were mixed in a 5:1 molar ratio and HDI was performed to enable a transposon-mediated stable intrahepatic gene transfer and development of autochthonous liver cancers, as described previously [3-6, 9]. All reagents used in the study are listed in Supplementary Table S4.

***Induction of liver fibrosis***

To induce liver fibrosis, C57BL/6J mice were treated as previously described [12, 13]. Briefly, six weeks old mice were treated with 4 µl/g body weight of 10% CCl_4_ (Sigma Aldrich, St. Louis, Missouri, USA) solution diluted in sunflower oil (Sigma Aldrich, St. Louis, Missouri, USA) for eight weeks (*i.p.* twice per week). The CCl_4_ treatment was continued after the vaccination. Control mice were treated with NaCl or sunflower oil (4 µl/g body weight).

***Vaccine strains and vaccination of mice***

Vaccine strains LmAI (DP6054) and LmAIO (DP6128) were provided by Prof. Dr. Daniel A. Portnoy (Department of Molecular & Cell Biology, University of California, Berkeley, CA, USA) and generation of double-deficient Listeria strains was described previously [14]. The genetic engineering work with these Listeria strains was approved by respective German authorities (accession numbers 40654/3/41 and 40611/0901/626).

For preparation of 0.1 LD_50_ vaccination dose of LmAI and LmAIO, overnight cultures grown in brain heart infusion broth (BHI, Becton Dickinson, Franklin Lakes, New Jersey, USA) supplemented with chloramphenicol were transferred into fresh broth and incubated at 37°C, 180 rpm for three hours. After reaching the logarithmic growth phase, bacteria were harvested by centrifugation and pellets were washed twice with pre-chilled sterile PBS, re-suspended in PBS and adjusted to 1 x 10^8^ CFU/ml. For vaccination of mice 100 µl of the LmAI or LmAIO suspensions (1 x 10^7^ CFU) were injected *i.v.*, whereas control mice received either 100 µl of sterile PBS *i.v.* or 50 µg of LPS-free Ova protein (EndoGrade Ovalbumin, lyophilized, Hyglos, Bernried am Starnberger See, Germany) diluted in PBS and administered *i.p.* For subsequent exact determination of CFU, different dilutions of the bacterial suspensions were plated on BHI agar and CFU counts were quantified after incubation at 37°C. All reagents used in the study are listed in Supplementary Table S4.

***Bacterial burden***

At sampling and for dose control 24 hours after vaccination mice were euthanized and blood was collected from the retro-orbital plexus and diluted 1:1 with sterile heparin 50 units (Ratiopharm GmbH, Ulm, Germany). Liver, spleen, pancreas and lung were isolated aseptically. Ipegal (Sigma Aldrich, St. Louis, Missouri, USA) solution (diluted in PBS/0.2%) was added and organs were homogenized. Different dilutions thereof were plated on BHI agar with respective antibiotics to quantify bacterial CFU. All reagents used in the study are listed in Supplementary Table S4.

***Tumor quantification***

Mice were dissected either at defined time points or after reaching a critical tumor burden. Livers were carefully removed, photographed, and all visible tumor nodules on the organ surface were quantified. Livers carrying an uncountable number of malignant lesions (too numerous to count, TNTC) were considered to exhibit 100 nodules. Liver samples were snap-frozen in liquid nitrogen or fixed in 4% paraformaldehyde (PFA, Sigma Aldrich, St. Louis, Missouri, USA) for subsequent histological examination. All reagents used in the study are listed in Supplementary Table S4.

***Leukocytes isolation, staining, flow cytometry analysis and sorting***

Leukocytes were isolated from livers, liver-draining portal LNs, spleens and blood as previously described [5, 6, 9]. Blood was collected from retro-orbital plexus and mixed with 50 units of heparin (Ratiopharm GmbH, Ulm, Germany). The obtained single cell suspensions from spleen, liver and LNs were blocked with anti-CD16/32 (clone 93 Biolegend, California, San Diego, USA). Surface and intracellular receptors were stained using established protocols [5, 6, 9] with anti-CD19 (clone 1D3, BD Bioscience, Franklin Lake, New Jersey, USA), anti-MHCII (clone M5/114.15.2, eBioscience, San Diego, USA), anti-CD80 (clone 16-10A1, Biolegend, California, San Diego, USA), anti-B220 (clone RA3-6B2, Biolegend, California, San Diego, USA), anti-F4/80 (clone BM8, Biolegend, California, San Diego, USA), anti-CD3 (clone 145-2C11, Biolegend, California, San Diego, USA), anti-CD4 (clone GK1.5, Biolegend, California, San Diego, USA), anti-CD8 (clone 53-6.7, Biolegend, California, San Diego, USA), anti-CD11 (clone N418, Biolegend, California, San Diego, USA), anti-CD11b (clone M1/70, Biolegend, California, San Diego, USA), anti-CD68 (clone FA-11, Biolegend, California, San Diego, USA), anti-Foxp3 (clone FJK-16s, eBioscience, San Diego, USA), anti-PD-1 (clone RMP1-30, Biolegend, California, San Diego, USA), anti-CD160 (clone 7H1, Biolegend, California, San Diego, USA), anti-LAG3 (clone C9B7W, Biolegend, California, San Diego, USA), and anti-4-1BBL (clone TKS-1, Biolegend, California, San Diego, USA) antibodies, respectively, and analyzed using a flow cytometer (LSRII SORP, BD Biosciences, Franklin Lake, New Jersey, USA). Detailed gating strategies are depicted in Supplementary Figure 7A, K, 8C-D. Data analyses were performed using FlowJo software (Tree Star).

For cell sorting, cells were isolated from spleen, liver-draining portal LNs and livers of C57BL/6-Foxp3^tm1Flv^/J transgenic mice [7], in which Foxp3^+^ Tregs can be tracked and thereby excluded using red fluorescent protein (RFP^+^). The obtained cell suspensions were stained with anti-CD3 (clone 145-2C11, Biolegend, California, San Diego, USA), anti-CD4 (clone RM4–5, Biolegend, California, San Diego, USA), anti-CD8 (clone 53-6.7, Biolegend, California, San Diego, USA), anti-CD44 (clone IM7, Biolegend, California, San Diego, USA) and anti-NK1.1 (clone PK136, Biolegend, California, San Diego, USA) antibodies and subjected to one-step cell sorting on a MOFlo (DakoCytomation, Glostrup, Denmark) or a FACSAria (BD Biosciences, Franklin Lakes, New Jersey, USA) sorter. The sorted Foxp3^-^ NK1.1^-^ CD3^+^ CD4^+^ CD44^+^ and Foxp3^-^ NK1.1^-^ CD3^+^ CD8^+^ CD44^+^ T lymphocytes were found to be of 98% purity. All antibodies used in the study are listed in Supplementary Table S6.

***Histopathology and (immune)histochemistry of formalin-fixed and paraffin-embedded samples***

Tissue samples were fixed in 4% PFA at room temperature for 24-48 hours and paraffin-embedded. Tissue sections were prepared using a routine microtome. Staining with hematoxylin and eosin (H&E), Elastica van Giesson (reticulin staining), anti-CK7 (EPR17078, 1:1000 diluted, abcam, Cambridge, UK),) anti-arginase 1 (N/A, 1:250 diluted, Zytomed Systems, Bargteheide, Germany) and anti-*Listeria monocytogenes* (Serotype 4b, 1:1000 diluted, abcam, Cambridge, UK) were performed for histopathological evaluation by experienced pathologists as described previously [15, 16]. Sirius red-staining in livers [3] of fibrosis-bearing mice upon CCl_4_ treatment and histopathological evaluation of fibrotic scars (Ishak score) were performed by experienced pathologists. Microscopic analyses were performed using standard light-microscopy (BX51, Olympus). All antibodies used in the study are listed in Supplementary Table S6.

***Determination of enzyme activities in plasma***

Blood was collected from retro-orbital plexus and mixed with 50 units of heparin (Ratiopharm GmbH, Ulm, Germany). Plasma was prepared by centrifugation at 400 x g for 10 minutes at 4°C and stored at -80°C. The enzyme activities of ALT and AST were measured on a cobas® 8000 modular automatic analyzer system using standard methods (Roche Diagnostics, Mannheim, Germany): ALTPM and ASTPM as established [17]. ALT and AST activity was expressed in U/l.

***ELISPOT***

To assess antigen-specific immune responses, splenocytes were isolated from C57BL/6J WT or p19^Arf-/-^ mice at sampling. The number of cells secreting IL-2, 4, 10, 17, or IFN-ɣ were determined using the corresponding detection kits from BD Bioscience (Franklin Lake, New Jersey, USA). Responses were determined following re-stimulation of 4 x 10^5^ cells/well with 5 µg/ml Ova peptides Ova_265-280_ TEWTSSNVMEERKIKV and Ova_257-264_ SIINFEKL, 5 µg/ml mutated RAS^G12V^ peptides (NRAS^G12V^_2-16_ TEYKLVVVGAVGVGK and NRAS^G12V^_7-21_ VVVGAVGVGKSALTI), 5 µg/ml Ova protein (EndoGrade Ovalbumin, lyophilized, Hyglos, Bernried am Starnberger See, Germany) or 1 x 10^7^ heat-killed LmAI or LmAIO as whole cell antigen. For the latter one, LmAI or LmAIO were heat-inactivated for 1 hour at 80°C and subsequently sonicated for 15 minutes on ice (duty cycle 90, output control 5, Branson sonifier 250, Branson Ultrasonics, Panbury, Connecticut, USA). Untreated (negative control) and concanavalin A-stimulated cells (positive control) served as controls (concanavalin A, Sigma Aldrich, St. Louis, Missouri, USA). After co-incubation, cells were lysed with Milli Q water and plates were processed according to the manufacturer’s instructions. Spots representing cytokine-producing cells were quantified with the ELISPOT plate reader (Immunospot, CTL, Shaker Heights, Ohio, USA). All antibodies/reagents used for ELISPOT assays are listed in Supplementary Table S4.

***ELISA***

Antigen-specific IgM and IgG antibodies were analyzed in serum/plasma of mice according to standard ELISA protocols described previously [18-20]. Briefly, flat-bottomed 96-well ELISA plates were coated overnight with the corresponding antigen dissolved in carbonate buffer (2 µg/ml Ova protein (EndoGrade Ovalbumin, lyophilized, Hyglos, Bernried am Starnberger See, Germany), or LmAI or LmAIO whole cell antigen (see “ELISPOT” section for the preparation details)). Thereafter, plates were blocked with 3% BSA/PBS and supplied afterwards with serum for 1 hour at 37°C. Detection was performed using anti-mouse IgM-biotin or anti-mouse IgG-biotin antibodies (both Sigma Aldrich, St. Louis, Missouri, USA) followed by incubation with streptavidin-conjugated horseradish peroxidase (HRP, BD Pharmingen, Franklin Lakes, New Jersey, USA). HRP substrate solution 2,2'-azino-bis (3-ethylbenzothiazoline-6-sulphonic acid) (ABTS, Sigma Aldrich, St. Louis, Missouri, USA) was added for detection of bound IgG/IgM. Samples were measured at optical density of 405 nm (OD_405_) using a Synergy 2 microplate reader (BioTek, Winooski, Vermont, USA). All samples were examined in triplicates. Software Gen5 was used for data analysis. All reagents used for ELISA analyses are listed in Supplementary Table S4.

***Combination treatment with α-CD20***

HCC-Ova was induced in p19^Arf-/-^ mice using HDI as described above. 7 days after HDI, mice were administered with LmAIO vaccine or respective controls. Ova-specific IgGs were monitored in plasma of mice overtime using ELISA (as described above). As soon as Ova-specific IgGs reached 0.09 (OD_405_), B cell-depletion therapy was started and mice were treated with 250 µg α-CD20 antibody *i.p*. twice per week, as described [21, 22]. All antibodies used in the study are listed in Supplementary Table S4.

***Combination treatment with α-PD-1***

HCC-Ova was induced in p19^Arf-/-^ mice using HDI as described above. 24 days (3.5 weeks) after HDI, mice were vaccinated in a weekly interval with 1 x 10^7^ CFU of LmAIO. For combination therapy, mice were treated with 300 µg α-PD-1 antibody (Biolegend, San Diego, California, USA) *i.p.* every 2-3 days five weeks long. Control groups received PBS or single therapy regime. All antibodies used in this study are listed in Supplementary Table S6.

***Transcriptome analyses (microarray)***

T lymphocytes were isolated using sorting from livers, liver-draining portal LNs and spleens of HCC-bearing C57BL/6-Foxp3^tm1Flv^/J mice with stable intrahepatic overexpression of *NRAS^G12V^* and c-*Myc* oncogenes, delivered via HDI. T lymphocytes from HCC-free mice, expressing only one oncogene (*NRAS^G12V^* or *c-Myc*) served as controls, C1 and C2, respectively.

Total RNA from sorted Foxp3^-^ NK1.1^-^ CD3^+^ CD4^+^ CD44^+^ and Foxp3^-^ NK1.1^-^ CD3^+^ CD8^+^ CD44^+^ T lymphocytes was isolated using the RNeasy kit (Qiagen, Hilden, Germany). The quality and integrity of the total RNA was controlled by means of an Agilent Technologies 2100 Bioanalyzer (Agilent Technologies, Waldbronn, Germany). Thereafter, 500 ng of total RNA was used for a Cy3-labelling reaction with the one-color Quick Amp Labeling Kit (Agilent Technologies, Waldbronn, Germany). Labeled cRNA was hybridized to Agilent’s mouse 4 x 44k microarrays for 16 h at 68°C. The latter were scanned using the Agilent DNA Microarray Scanner. Two replicates in total were analyzed. Expression values were calculated with the Feature Extraction v10.7.3.1 software package (Agilent Technologies, Waldbronn, Germany).

The obtained data were further analyzed using R package “limma” [23]. Raw data were log2 transformed and quantile normalized. For testing differential gene expression, normalized data sets were filtered for informative genes (showing at least expression values > log2 (50) in more than two samples). For statistical analysis and assessing differential expression among tumor-bearing and control mice, limma uses an empirical Bayes method to moderate the standard errors of the estimated log-fold changes. All antibodies and software used in the study are listed in Supplementary Tables S6 and S7. The raw data has been deposited in the Gene Expression Omnibus (GEO) database, see “Data availability” section below.

***Statistics***

To calculate significance of survival analysis, Mantel-Cox test was used. The unpaired Student’s *t*-test was used for all other statistical analyses to calculate significant differences among experimental and control groups. The experiments generally were repeated two to four times. The number of animals used for the experiments is depicted on plots and/or in figure legends. If not stated otherwise, minimal 4-5 mice per group were used. Statistical analysis of microarray data using Bayes method is described in the section “Transcriptome analyses (microarray)”. If not stated otherwise, data are shown as mean +/- standard error of the mean (SEM) with *P < 0.05* considered statistically significant. Significance levels were denoted as: **P* < 0.05, ***P* < 0.01, ****P* < 0.001 and *****P* < 0.0001.

***Data availability***

All data associated with this study are present in this paper or in the Supplementary Information. Data obtained from the microarray has been deposited in the GEO expression database (http://www.ncbi.nlm.nih.gov/geo/) under the accession number GSE144811.

**Supplementary Table S3. Murine strains used in the study.**

| **Name** | **Strain** | **Supplier** | **Gender** | **Age** | **Citation** |
| --- | --- | --- | --- | --- | --- |
| Mouse | C57BL/6J | Charles River | Males and females | 4-8 weeks at HDI |  |
| Mouse | B6.129X1-Cdkn2a^tm1Cjs^ (p19^Arf-/-^) | HZI | Males and females | 4-8 weeks at HDI | [1] |
| Mouse | C57BL/6-Foxp3^tm1Flv^/J | Jackson Laboratory | Males and females | 6-8 weeks at HDI | [7] |

**Supplementary Table S4. Reagents.**

| **Reagent or Resource** | **Supplier / Citation** | **Identifier** |
| --- | --- | --- |
| SB13 vector | [24] |  |
| CaKIO vector | This study |  |
| CaMIG vector | This study |  |
| CaN vector | [3, 5, 6, 9] |  |
| CaNIO vector | [6] |  |
| Mouse IFN-ɣ ELISPOT Pair | BD Bioscience | Cat # 551881 |
| Mouse IL-2 ELISPOT Pair | BD Bioscience | Cat # 551876 |
| Mouse IL-4 ELISPOT Pair | BD Bioscience | Cat # 551878 |
| Anti-Mouse IL-10 (Capture) | eBioscience | Cat # 16-7101-85 |
| Anti-Mouse IL-10 (Detection) | eBioscience | Cat # 13-7102-85 |
| Anti-Mouse IL-17A (Capture) | eBioscience | Cat # 16-7175-85 |
| Anti-Mouse IL-17A (Detection) | eBioscience | Cat # 13-7177-85 |
| HRP Streptavidin | eBioscience | Cat # 557630 |
| IgG Biotin Conjugated | Sigma-Aldrich | Cat # B-7022 |
| IgM Biotin Conjugated | Sigma-Aldrich | Cat # B-9265 |
| Streptavidin-HRP | Pharmingen | Cat # 554066 |
| EndoGrade Ovalbumin | Hyglos | Cat # 32100 |
| QIAGEN EndoFree Maxi Kit | Qiagen | Cat # 12362 |
| Brain Heart Infusion | BD | Cat # 237200 |
| Igepal CA-630 | Sigma Aldrich | Cat # I8896 |
| Concanavalin A | Sigma Aldrich | Cat # C5275 |
| Heparin 5000 | Ratiopharm GmbH | PZN-03029820 |
| CCl_4_ | Sigma Aldrich | Cat # 1098295 |
| Sunflower Oil | Sigma Aldrich | Cat # 8001-21-6 |
| Chloramphenicol | Sigma Aldrich | Cat # C0378 |

**Supplementary Table S5. DNA sequences.**

| **Name** | **Sequence** | **Supplier** |
| --- | --- | --- |
| KRAS MluI fw | GGAGGAACGCGTATGACTGAGTATAAACTTG | Eurofins |
| KRAS NotI rv | TGTAGCGGCCGCGTATTCACATAACTGTACACC | Eurofins |
| IRES NotI fw | GGAGGAGCGGCCGCATCGAGGTTAACGAATTCCGCCC | Eurofins |
| Ova AgeI rv | TCGCCAACCGGTTTAAGGGGAAACACATCTGC | Eurofins |

**Supplementary Table S6. Antibodies.**

| **Name** | **Supplier** | **Cat no.** | **Clone no.** | **Citation** |
| --- | --- | --- | --- | --- |
| Anti-CD16/32 | Biolegend | 101320 | 93 | [25] |
| Anti-CD19 | BD Bioscience | 553786 | 1D3 | [26] |
| Anti-MHCII | eBioscience | 13-5321-82 | M5/114.15.2 | [27] |
| Anti-Foxp3 | eBioscience | 14-5773-80 | FJK-16s | [28] |
| Anti-CD80 | Biolegend | 104731 | 16-10A1 | [29] |
| Anti-CD68 | Biolegend | 137007 | FA-11 | [30] |
| Anti-B220 | Biolegend | 103236 | RA3-6B2 | [31] |
| Anti-F4/80 | Biolegend | 123115 | BM8 | [32] |
| Anti-CD3 | Biolegend | 100222 | 17A2 | [33] |
| Anti-PD-1 | Biolegend | 109105 | RMP1-30 | [34] |
| Anti-CD4 | Biolegend | 100430 | GK1.5 | [35] |
| Anti-CD8 | Biolegend | 100761 | 53-6.7 | [36] |
| Anti-CD11c | Biolegend | 117347 | N418 | [37] |
| Anti-CD11b | Biolegend | 101243 | M1/70 | [38] |
| Anti-CD160 | Biolegend | 143003 | 7H1 | [39] |
| Anti-LAG3 | Biolegend | 125219 | C9B7W | [40] |
| Anti-4-1BBL | Biolegend | 107103 | TKS-1 | [41] |
| Anti-PD-1 | Biolegend | 135219 | 29F.1A12 | [42] |
| Anti-CD44 | Biolegend | 103044 | IM7 | [43] |
| Anti-NK1.1 | Biolegend | 108731 | PK136 | [44] |
| Ultra-LEAF purified anti-PD-1 | Biolegend | 114119 | RMP1-14 | [45] |
| Anti-CK-7 | Abcam | ab181598 | EPR17078 | [46] |
| Anti-arginase 1 | Zytomed Systems | 501-19282 | polyclonal | Not available |
| Anti-*Listeria monocytogenes* | Abcam | ab35132 | polyclonal | [47] |
| Ultra-LEAF purified anti-CD20 | Biolegend | SA271G2 | monoclonal | [48] |

**Supplementary Table S7. Software.**

| **Software name** | **Manufacturer** | **Version** |
| --- | --- | --- |
| FlowJo | Treestar Inc. | **9.9.6** |
| Graphpad prism | Graphpad Software, Inc. | **5** |
| R package limmaGUI | Smyth |  |
| BD Accuri C6 | BD | **6** |
| Software Gen5 | Biotek | **1.06.10** |

**Supplementary References**

1. Kamijo T, Zindy F, Roussel MF, Quelle DE, Downing JR, Ashmun RA, et al. Tumor suppression at the mouse INK4a locus mediated by the alternative reading frame product p19ARF. *Cell* 1997;91(5):649-59.

2. Rudalska R, Dauch D, Longerich T, McJunkin K, Wuestefeld T, Kang TW, et al. In vivo RNAi screening identifies a mechanism of sorafenib resistance in liver cancer. *Nat Med* 2014;20(10):1138-46.

3. Dauch D, Rudalska R, Cossa G, Nault JC, Kang TW, Wuestefeld T, et al. A MYC-aurora kinase A protein complex represents an actionable drug target in p53-altered liver cancer. *Nat Med* 2016;22(7):744-53.

4. Seehawer M, Heinzmann F, D'Artista L, Harbig J, Roux PF, Hoenicke L, et al. Necroptosis microenvironment directs lineage commitment in liver cancer. *Nature* 2018;562(7725):69-75.

5. Petriv N, Neubert L, Vatashchuk M, Timrott K, Suo H, Hochnadel I, et al. Increase of alpha-dicarbonyls in liver and receptor for advanced glycation end products on immune cells are linked to nonalcoholic fatty liver disease and liver cancer. *Oncoimmunology* 2021;10(1):1874159.

6. Kang TW, Yevsa T, Woller N, Hoenicke L, Wuestefeld T, Dauch D, et al. Senescence surveillance of pre-malignant hepatocytes limits liver cancer development. *Nature* 2011;479(7374):547-51.

7. Wan YY, Flavell RA. Identifying Foxp3-expressing suppressor T cells with a bicistronic reporter. *Proc Natl Acad Sci U S A* 2005;102(14):5126-31.

8. Carlson CM, Frandsen JL, Kirchhof N, McIvor RS, Largaespada DA. Somatic integration of an oncogene-harboring Sleeping Beauty transposon models liver tumor development in the mouse. *Proc Natl Acad Sci U S A* 2005;102(47):17059-64.

9. Eggert T, Wolter K, Ji J, Ma C, Yevsa T, Klotz S, et al. Distinct Functions of Senescence-Associated Immune Responses in Liver Tumor Surveillance and Tumor Progression. *Cancer Cell* 2016;30(4):533-47.

10. Gurlevik E, Fleischmann-Mundt B, Brooks J, Demir IE, Steiger K, Ribback S, et al. Administration of Gemcitabine After Pancreatic Tumor Resection in Mice Induces an Antitumor Immune Response Mediated by Natural Killer Cells. *Gastroenterology* 2016;151(2):338-50 e7.

11. Gurlevik E, Fleischmann-Mundt B, Armbrecht N, Longerich T, Woller N, Kloos A, et al. Adjuvant gemcitabine therapy improves survival in a locally induced, R0-resectable model of metastatic intrahepatic cholangiocarcinoma. *Hepatology* 2013;58(3):1031-41.

12. Song G, Pacher M, Balakrishnan A, Yuan Q, Tsay HC, Yang D, et al. Direct Reprogramming of Hepatic Myofibroblasts into Hepatocytes In Vivo Attenuates Liver Fibrosis. *Cell Stem Cell* 2016;18(6):797-808.

13. Wuestefeld T, Pesic M, Rudalska R, Dauch D, Longerich T, Kang TW, et al. A Direct in vivo RNAi screen identifies MKK4 as a key regulator of liver regeneration. *Cell* 2013;153(2):389-401.

14. Brockstedt DG, Giedlin MA, Leong ML, Bahjat KS, Gao Y, Luckett W, et al. Listeria-based cancer vaccines that segregate immunogenicity from toxicity. *Proc Natl Acad Sci U S A* 2004;101(38):13832-7.

15. Neubert L, Borchert P, Stark H, Hoefer A, Vogel-Claussen J, Warnecke G, et al. Molecular Profiling of Vascular Remodeling in Chronic Pulmonary Disease. *Am J Pathol* 2020;190(7):1382-96.

16. Neubert L, Borchert P, Shin HO, Linz F, Wagner WL, Warnecke G, et al. Comprehensive three-dimensional morphology of neoangiogenesis in pulmonary veno-occlusive disease and pulmonary capillary hemangiomatosis. *J Pathol Clin Res* 2019;5(2):108-14.

17. Lichtinghagen R, Pietsch D, Bantel H, Manns MP, Brand K, Bahr MJ. The Enhanced Liver Fibrosis (ELF) score: normal values, influence factors and proposed cut-off values. *J Hepatol* 2013;59(2):236-42.

18. Ebensen T, Libanova R, Schulze K, Yevsa T, Morr M, Guzman CA. Bis-(3',5')-cyclic dimeric adenosine monophosphate: strong Th1/Th2/Th17 promoting mucosal adjuvant. *Vaccine* 2011;29(32):5210-20.

19. Yevsa T, Ebensen T, Fuchs B, Zygmunt B, Libanova R, Gross R, et al. Development and characterization of attenuated metabolic mutants of Bordetella bronchiseptica for applications in vaccinology. *Environ Microbiol* 2013;15(1):64-76.

20. Schneider C, Teufel A, Yevsa T, Staib F, Hohmeyer A, Walenda G, et al. Adaptive immunity suppresses formation and progression of diethylnitrosamine-induced liver cancer. *Gut* 2012;61(12):1733-43.

21. Shalapour S, Lin XJ, Bastian IN, Brain J, Burt AD, Aksenov AA, et al. Inflammation-induced IgA+ cells dismantle anti-liver cancer immunity. *Nature* 2017;551(7680):340-5.

22. DiLillo DJ, Hamaguchi Y, Ueda Y, Yang K, Uchida J, Haas KM, et al. Maintenance of long-lived plasma cells and serological memory despite mature and memory B cell depletion during CD20 immunotherapy in mice. *J Immunol* 2008;180(1):361-71.

23. Smyth GK. Linear models and empirical bayes methods for assessing differential expression in microarray experiments. *Stat Appl Genet Mol Biol* 2004;3:Article3.

24. Yant SR, Park J, Huang Y, Mikkelsen JG, Kay MA. Mutational analysis of the N-terminal DNA-binding domain of sleeping beauty transposase: critical residues for DNA binding and hyperactivity in mammalian cells. *Mol Cell Biol* 2004;24(20):9239-47.

25. Minkah N, Macaluso M, Oldenburg DG, Paden CR, White DW, McBride KM, et al. Absence of the uracil DNA glycosylase of murine gammaherpesvirus 68 impairs replication and delays the establishment of latency in vivo. *J Virol* 2015;89(6):3366-79.

26. Sato S, Ono N, Steeber DA, Pisetsky DS, Tedder TF. CD19 regulates B lymphocyte signaling thresholds critical for the development of B-1 lineage cells and autoimmunity. *J Immunol* 1996;157(10):4371-8.

27. Bonacina F, Coe D, Wang G, Longhi MP, Baragetti A, Moregola A, et al. Myeloid apolipoprotein E controls dendritic cell antigen presentation and T cell activation. *Nat Commun* 2018;9(1):3083.

28. Martinez-Velez N, Garcia-Moure M, Marigil M, Gonzalez-Huarriz M, Puigdelloses M, Gallego Perez-Larraya J, et al. The oncolytic virus Delta-24-RGD elicits an antitumor effect in pediatric glioma and DIPG mouse models. *Nat Commun* 2019;10(1):2235.

29. Cousin C, Oberkampf M, Felix T, Rosenbaum P, Weil R, Fabrega S, et al. Persistence of Integrase-Deficient Lentiviral Vectors Correlates with the Induction of STING-Independent CD8(+) T Cell Responses. *Cell Rep* 2019;26(5):1242-57 e7.

30. Berry MR, Mathews RJ, Ferdinand JR, Jing C, Loudon KW, Wlodek E, et al. Renal Sodium Gradient Orchestrates a Dynamic Antibacterial Defense Zone. *Cell* 2017;170(5):860-74 e19.

31. Macal M, Tam MA, Hesser C, Di Domizio J, Leger P, Gilliet M, et al. CD28 Deficiency Enhances Type I IFN Production by Murine Plasmacytoid Dendritic Cells. *J Immunol* 2016;196(4):1900-9.

32. Patankar YR, Lovewell RR, Poynter ME, Jyot J, Kazmierczak BI, Berwin B. Flagellar motility is a key determinant of the magnitude of the inflammasome response to Pseudomonas aeruginosa. *Infect Immun* 2013;81(6):2043-52.

33. Draijer C, Robbe P, Boorsma CE, Hylkema MN, Melgert BN. Dual role of YM1+ M2 macrophages in allergic lung inflammation. *Sci Rep* 2018;8(1):5105.

34. Martinez-Lopez M, Iborra S, Conde-Garrosa R, Mastrangelo A, Danne C, Mann ER, et al. Microbiota Sensing by Mincle-Syk Axis in Dendritic Cells Regulates Interleukin-17 and -22 Production and Promotes Intestinal Barrier Integrity. *Immunity* 2019;50(2):446-61 e9.

35. Ma C, Han M, Heinrich B, Fu Q, Zhang Q, Sandhu M, et al. Gut microbiome-mediated bile acid metabolism regulates liver cancer via NKT cells. *Science* 2018;360(6391).

36. Ko SY, Ko HJ, Chang WS, Park SH, Kweon MN, Kang CY. alpha-Galactosylceramide can act as a nasal vaccine adjuvant inducing protective immune responses against viral infection and tumor. *J Immunol* 2005;175(5):3309-17.

37. Luck H, Khan S, Kim JH, Copeland JK, Revelo XS, Tsai S, et al. Gut-associated IgA(+) immune cells regulate obesity-related insulin resistance. *Nat Commun* 2019;10(1):3650.

38. Ponzetta A, Carriero R, Carnevale S, Barbagallo M, Molgora M, Perucchini C, et al. Neutrophils Driving Unconventional T Cells Mediate Resistance against Murine Sarcomas and Selected Human Tumors. *Cell* 2019;178(2):346-60 e24.

39. Wu J, Zhang H, Shi X, Xiao X, Fan Y, Minze LJ, et al. Ablation of Transcription Factor IRF4 Promotes Transplant Acceptance by Driving Allogenic CD4(+) T Cell Dysfunction. *Immunity* 2017;47(6):1114-28 e6.

40. Benci JL, Johnson LR, Choa R, Xu Y, Qiu J, Zhou Z, et al. Opposing Functions of Interferon Coordinate Adaptive and Innate Immune Responses to Cancer Immune Checkpoint Blockade. *Cell* 2019;178(4):933-48 e14.

41. Zhao Y, Croft M. Dispensable role for 4-1BB and 4-1BBL in development of vaccinia virus-specific CD8 T cells. *Immunol Lett* 2012;141(2):220-6.

42. Lindenstrom T, Moguche A, Damborg M, Agger EM, Urdahl K, Andersen P. T Cells Primed by Live Mycobacteria Versus a Tuberculosis Subunit Vaccine Exhibit Distinct Functional Properties. *EBioMedicine* 2018;27:27-39.

43. Burrack KS, Huggins MA, Taras E, Dougherty P, Henzler CM, Yang R, et al. Interleukin-15 Complex Treatment Protects Mice from Cerebral Malaria by Inducing Interleukin-10-Producing Natural Killer Cells. *Immunity* 2018;48(4):760-72 e4.

44. Dyer DP, Medina-Ruiz L, Bartolini R, Schuette F, Hughes CE, Pallas K, et al. Chemokine Receptor Redundancy and Specificity Are Context Dependent. *Immunity* 2019;50(2):378-89 e5.

45. Godoy-Calderon MJ, Gonzalez-Marcano E, Carballo J, Convit AF. Evaluation of a ConvitVax/anti-PD-1 combined immunotherapy for breast cancer treatment. *Oncotarget* 2019;10(61):6546-60.

46. Gao Q, Yang Z, Xu S, Li X, Yang X, Jin P, et al. Heterotypic CAF-tumor spheroids promote early peritoneal metastatis of ovarian cancer. *J Exp Med* 2019;216(3):688-703.

47. Sai K, Parsons C, House JS, Kathariou S, Ninomiya-Tsuji J. Necroptosis mediators RIPK3 and MLKL suppress intracellular Listeria replication independently of host cell killing. *J Cell Biol* 2019;218(6):1994-2005.

48. Chen YQ, Li PC, Pan N, Gao R, Wen ZF, Zhang TY, et al. Tumor-released autophagosomes induces CD4(+) T cell-mediated immunosuppression via a TLR2-IL-6 cascade. *J Immunother Cancer* 2019;7(1):178.
