# Supplementary figures and images for: Safety and efficacy of prophylactic and therapeutic vaccine based on live-attenuated Listeria monocytogenes in hepatobiliary cancers
Source: Oncogene. 2022 Feb 16;41(14):2039–53. doi: 10.1038/s41388-022-02222-z (PMC8853207; doi:10.1038/s41388-022-02222-z)

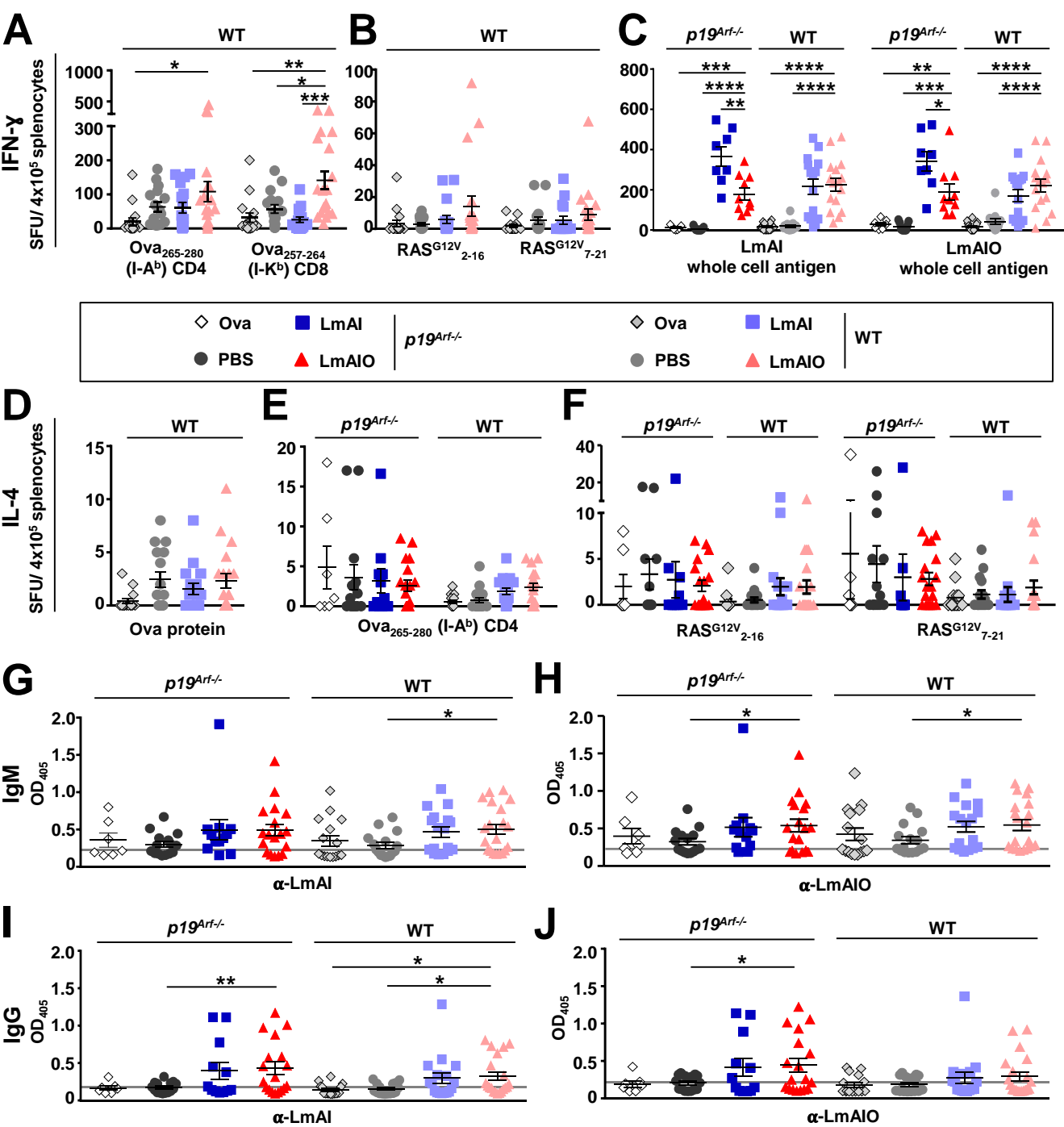

Fig. S2

**A**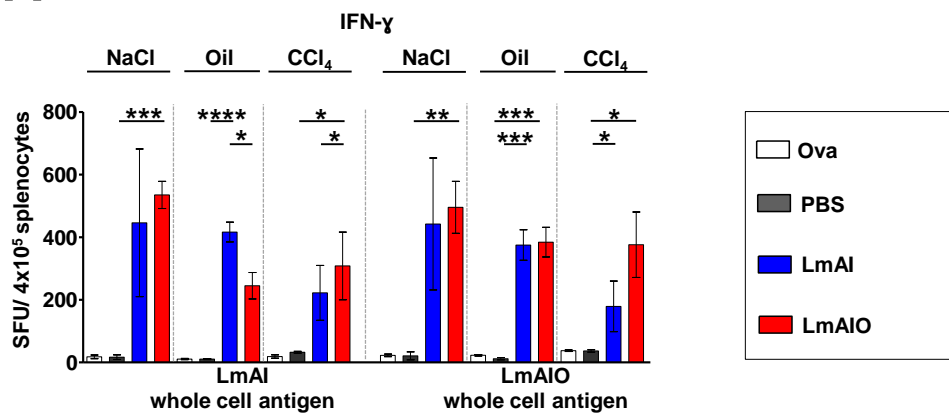**B**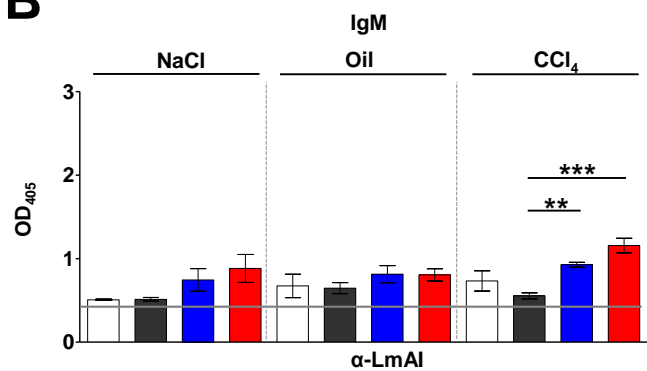**C**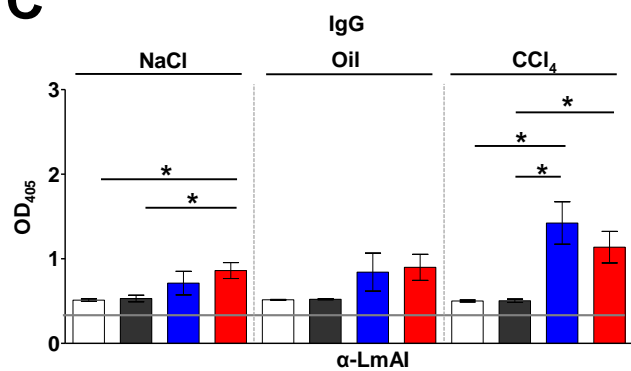**D**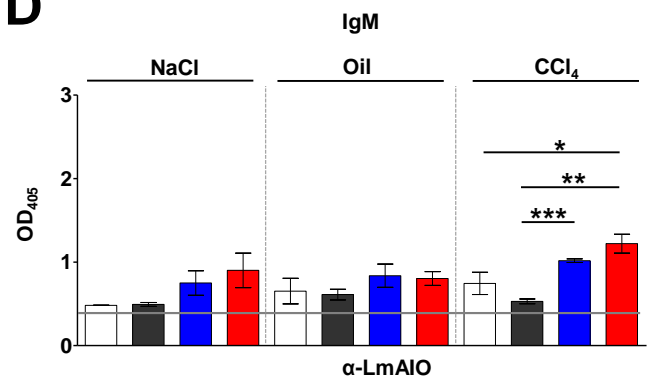**E**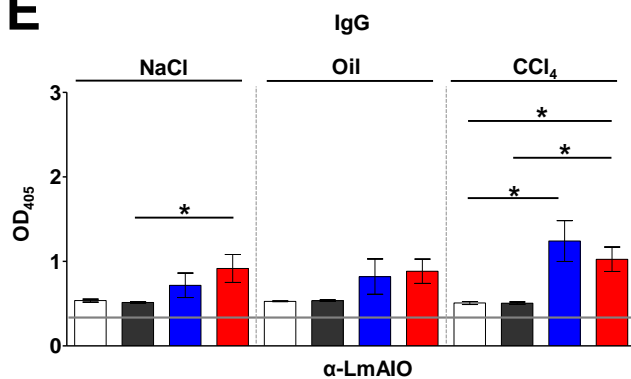**Fig. S3**

**A**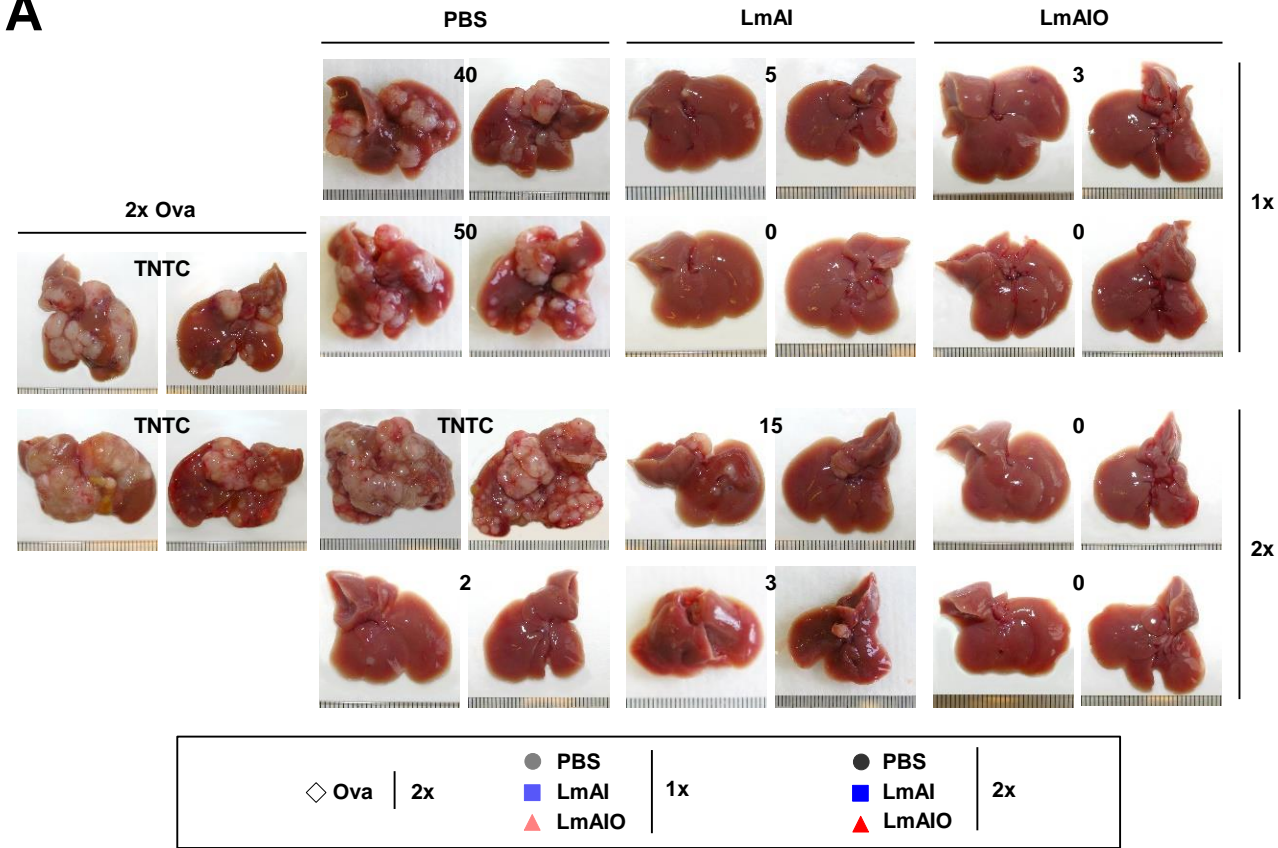**B**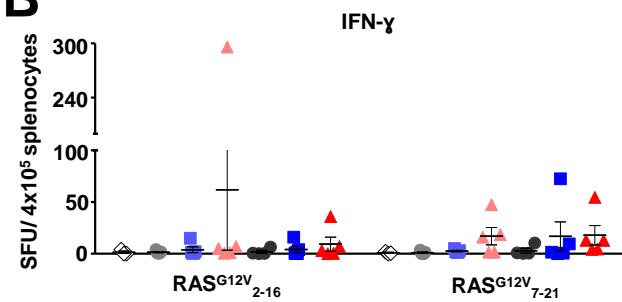**C**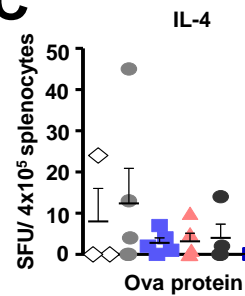**D**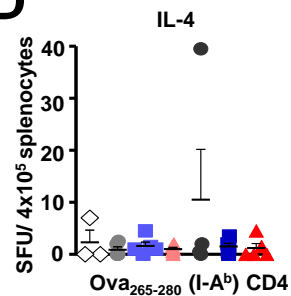**E**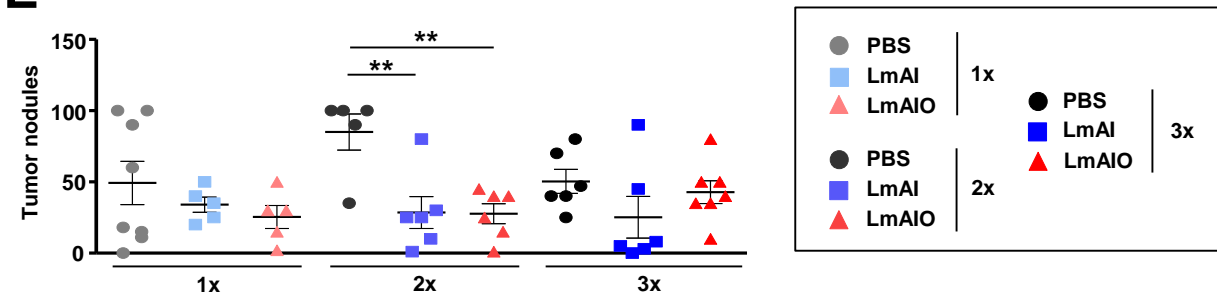**Fig. S4**

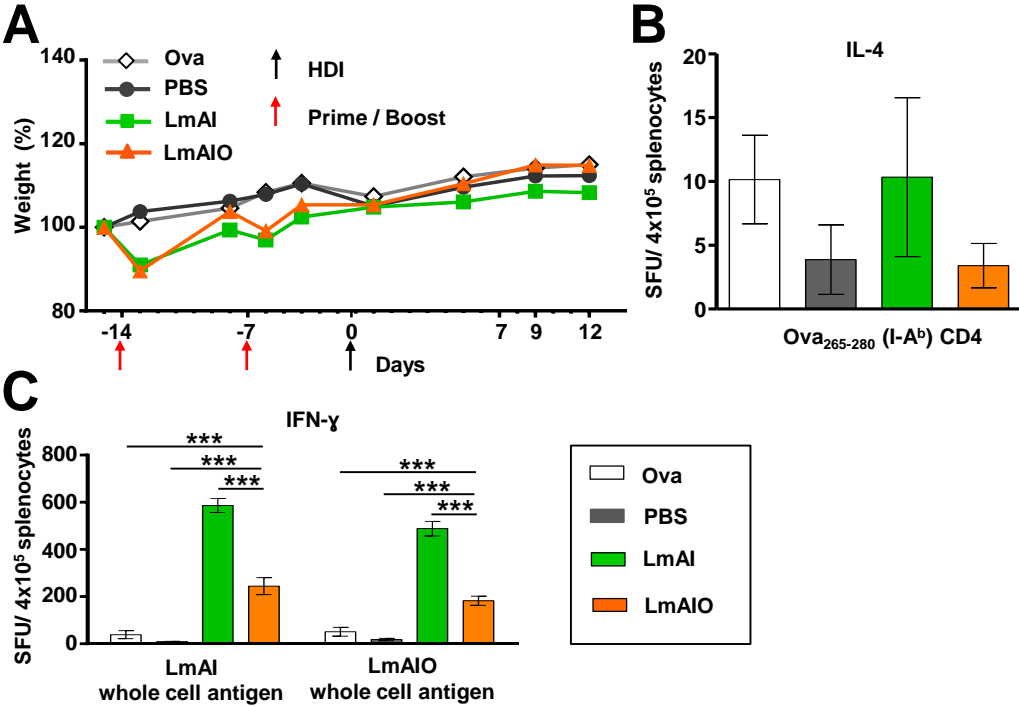

Fig. S5

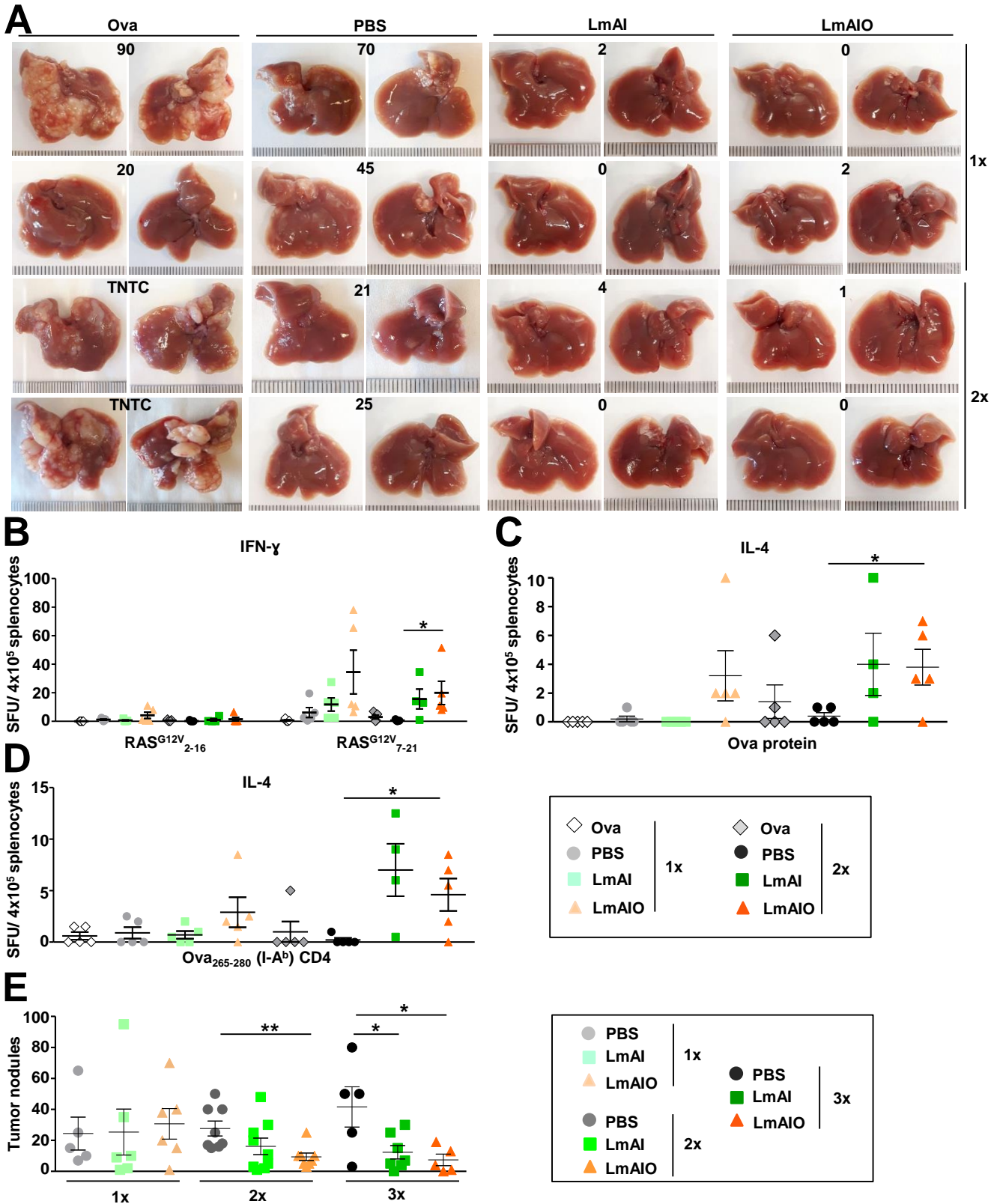

**Fig. S6**



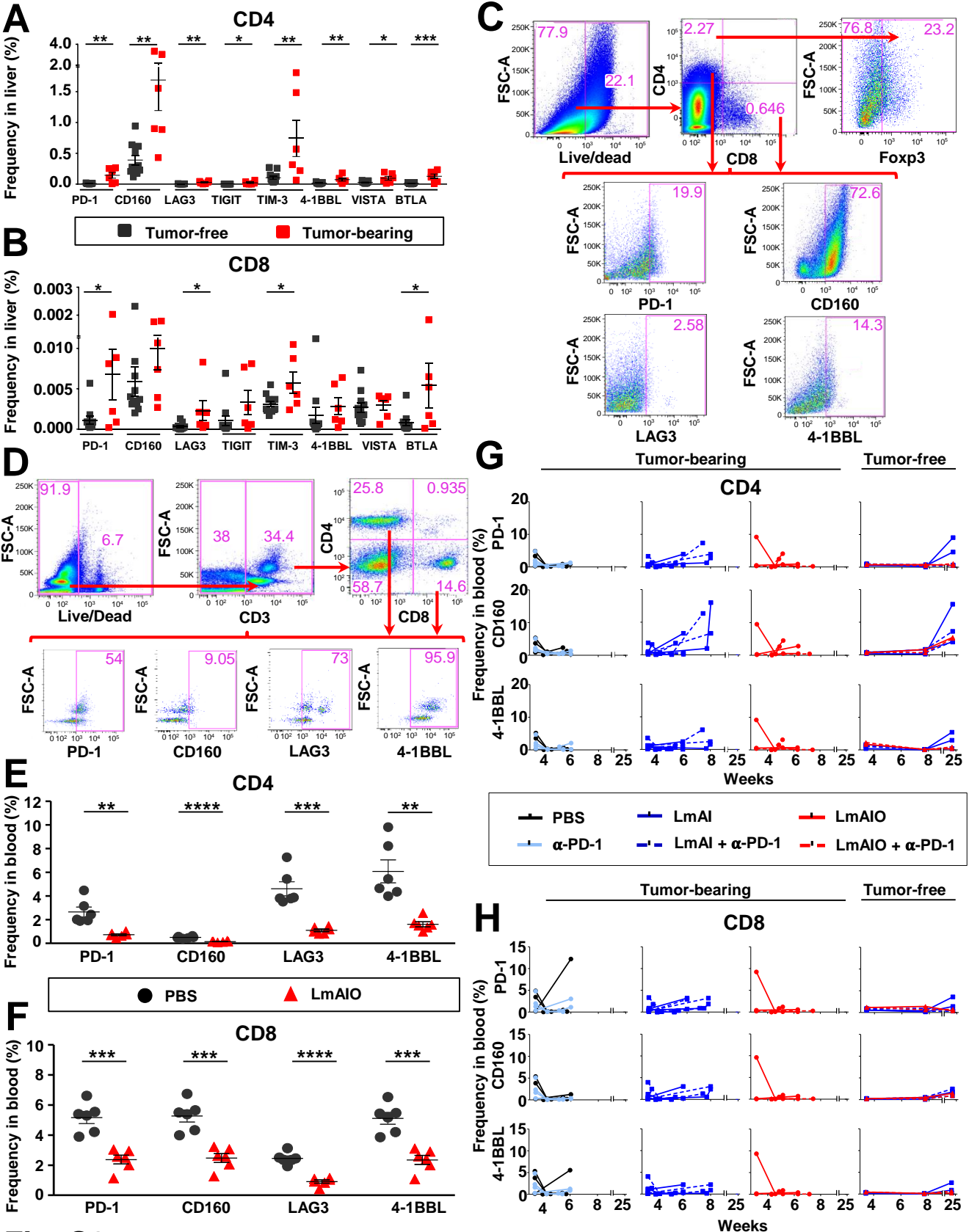

**Fig. S8**

Supplement: Supplementary file 2 — Supplementary Figures [file 41388_2022_2222_MOESM2_ESM.pdf]
